# The NMR contribution to protein–protein networking in Fe–S protein maturation

**DOI:** 10.1007/s00775-018-1552-x

**Published:** 2018-03-22

**Authors:** Lucia Banci, Francesca Camponeschi, Simone Ciofi-Baffoni, Mario Piccioli

**Affiliations:** 1 Magnetic Resonance Center CERM, University of Florence, Via Luigi Sacconi 6, Sesto Fiorentino, 50019 Florence, Italy; 2 Department of Chemistry, University of Florence, Via della Lastruccia 3, Sesto Fiorentino, 50019 Florence, Italy

**Keywords:** NMR spectroscopy, Hyperfine interactions, Fe–S proteins, Interactomics, CIA machinery, ISC machinery

## Abstract

Iron–sulfur proteins were among the first class of metalloproteins that were actively studied using NMR spectroscopy tailored to paramagnetic systems. The hyperfine shifts, their temperature dependencies and the relaxation rates of nuclei of cluster-bound residues are an efficient fingerprint of the nature and the oxidation state of the Fe–S cluster. NMR significantly contributed to the analysis of the magnetic coupling patterns and to the understanding of the electronic structure occurring in [2Fe–2S], [3Fe–4S] and [4Fe–4S] clusters bound to proteins. After the first NMR structure of a paramagnetic protein was obtained for the reduced *E. halophila* HiPIP I, many NMR structures were determined for several Fe–S proteins in different oxidation states. It was found that differences in chemical shifts, in patterns of unobserved residues, in internal mobility and in thermodynamic stability are suitable data to map subtle changes between the two different oxidation states of the protein. Recently, the interaction networks responsible for maturing human mitochondrial and cytosolic Fe–S proteins have been largely characterized by combining solution NMR standard experiments with those tailored to paramagnetic systems. We show here the contribution of solution NMR in providing a detailed molecular view of “Fe–S interactomics”. This contribution was particularly effective when protein–protein interactions are weak and transient, and thus difficult to be characterized at high resolution with other methodologies.

## Introduction

The NMR spectrum of cytochrome *c*, collected by Kowalsky in 1965, was the first high-resolution NMR spectrum of a paramagnetic protein published ever [1]. The hyperfine shifts induced onto the methyl resonances of heme by the paramagnetic Fe^3+^ ion were large enough to circumvent resolution problems and permitted, for the first time, the identification of “individual” proton resonances, which have been used as source of information on protein oxidation states and on the number and nature of heme ligands [2, 3]. Soon after that, NMR spectroscopy was applied on other paramagnetic proteins such as single iron rubredoxins [4] and on Fe–S cluster containing proteins [5, 6]. In combination with EPR, Mössbauer and magnetic susceptibility measurements, ^1^H NMR spectroscopy significantly contributed, since the early days of research on Fe–S proteins, to elucidate the electronic structure and magnetic coupling among the iron ions in Fe–S clusters [7–11]. Small electron transfer proteins such as rubredoxins, ferredoxins and HiPIPs are paradigmatic examples of how solution NMR can easily identify different types of Fe–S clusters and different oxidation states. Indeed, the number of iron ions, their oxidation states and the magnetic couplings among them determine NMR spectra that differ one another in terms of signal linewidths, chemical shifts and number of observed signals.

## Elucidation of electronic structure of Fe–S clusters in proteins

**Fig. 1:**
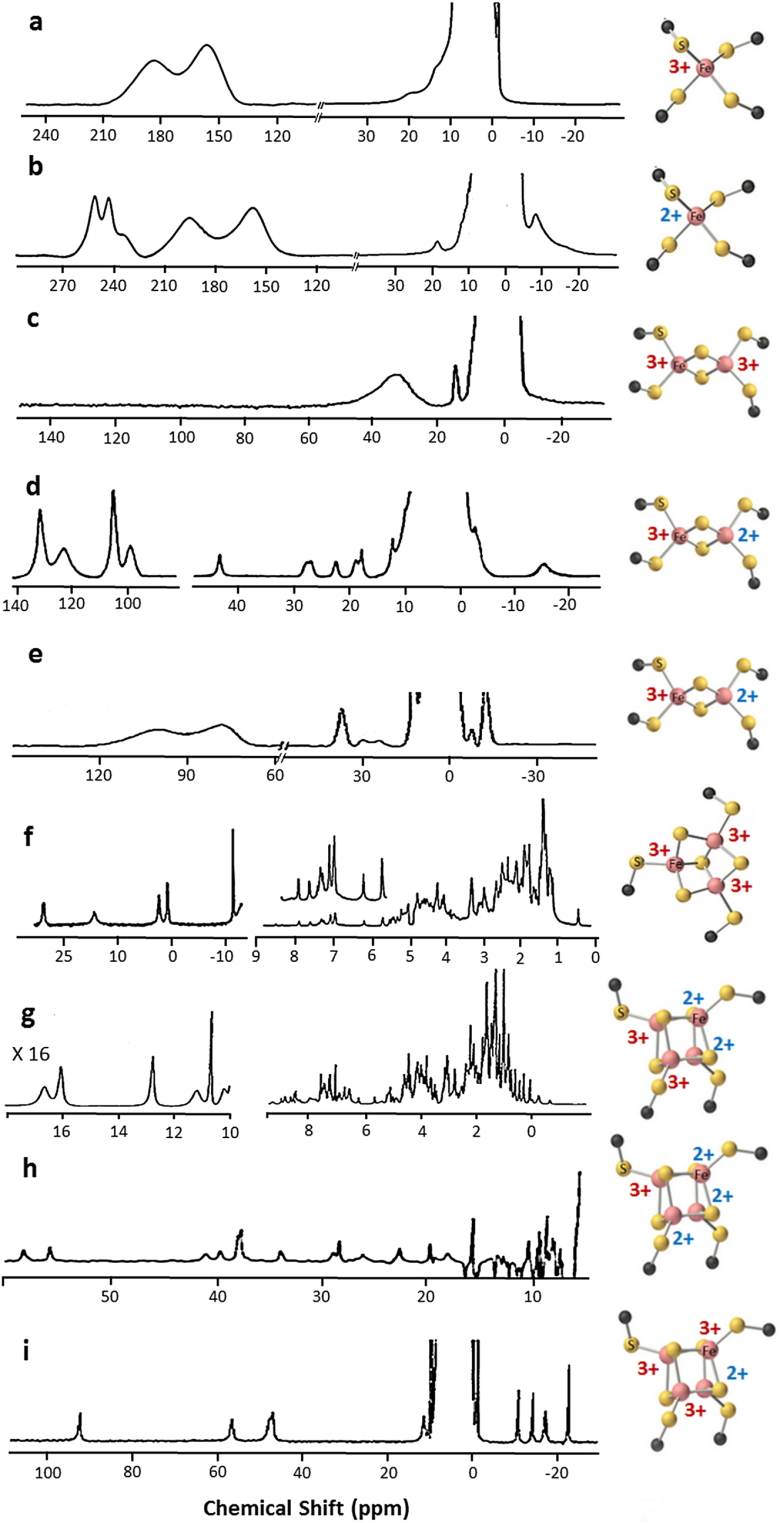
1D ^1^H NMR spectra of different Fe–S cluster types. 400 MHz 1D ^1^H NMR spectra of Fe^3+^ (**a**) and Fe^2+^ (**b**) *C. pasteurianum* rubredoxin, acquired at 308 K (adapted from [13]); **c** 200 MHz 1D ^1^H NMR spectrum of [2Fe–2S]^2+^ *P. umbilicalis* ferredoxin, acquired at 303 K [15]; **d** 360 MHz 1D ^1^H NMR spectrum of [2Fe–2S]^+^ *P. umbilicalis* ferredoxin, recorded at 303 K [25]; **e** 400 MHz 1D ^1^H NMR spectrum of [2Fe–2S]^+^ human ferredoxin, acquired at 303 K [201]; **f** 500 MHz 1D ^1^H NMR spectrum of [3Fe–4S]^+^ *P. furiosus* ferredoxin, recorded at 303 K [33]; 600 MHz 1D ^1^H NMR spectra of [4Fe–4S]^2+^, **g** [40] and [4Fe–4S]^3+^, **i** [60] *E. halophila* HIPIP II, recorded at 300 K; **h** 600 MHz 1D ^1^H NMR spectrum of [4Fe–4S]^+^ *C. acidi urici* ferredoxin, acquired at 298 K [43]

From the NMR spectroscopy point of view, there is a variety of possible behaviors and patterns. The least favorable situation is that occurring in oxidized single iron ion such as in rubredoxins (Fig. 1a). The first ^1^H NMR spectrum was reported by Moura and coworkers. Here an isolated, high spin Fe^3+^ ion (*S* = 5/2) gives a contribution to transverse nuclear relaxation rates of the βCH_2_ protons of iron-bound cysteines as large as 80 kHz [12]. Albeit their very fast nuclear relaxation rates, which determine large linewidths, Cys βCH_2_ signals are observable, thanks to their large hyperfine shifts, being well outside the diamagnetic envelope. The reduction of Fe^3+^ ion to high spin, *S* = 2, Fe^2+^, gives a significant decrease in the observed linewidths and an increase in the chemical shifts of the βCH_2_ resonances (Fig. 1b), which are, therefore, oxidation state-dependent spectral parameters [13, 14]. In this way, NMR can be exploited to obtain information on iron oxidation states in rubredoxins.

In the case of Fe–S clusters, the magnetic coupling between the iron ions determines various electron spin energy levels whose separation depends on the magnetic coupling constants [7, 15–18]. The coupling of the nuclear spins with these multiple electron spin levels significantly affect both the chemical shifts and the relaxation rates [15, 19, 20]. As a consequence of this coupling, NMR signals are sharper than those observed for isolated iron ions, and the contact shifts experienced by cluster-bound residues are usually smaller than those observed in rubredoxins. Consequently, the NMR spectra are dramatically different when changing oxidation state or cluster composition. The hyperfine shifts, their temperature dependencies and the relaxation rates of nuclei of cluster-bound residues allowed NMR spectroscopists to elucidate the magnetic coupling patterns occurring in [2Fe–2S], [3Fe–4S] and [4Fe–4S] clusters. This contributed to the understanding of the electronic structure of Fe–S clusters in proteins, also thanks to the fact that NMR provides information at room temperature. We would like to recall here that the Fe–S clusters are usually always characterized by only two redox states differing by a single electron. Let us briefly overview the different cases.

In [2Fe–2S] clusters, the oxidized [2Fe–2S]^2+^ state contains two antiferromagnetically coupled Fe^3+^ ions that give rise to a *S* = 0 ground state [21]. Antiferromagnetic coupling between two identical ions does not contribute significantly to reduce the electron spin relaxation times, and therefore, the βCH_2_ signals are quite broad [15, 22]. Their contact shifts are small compared to those in rubredoxins, in agreement with the fact that the contact shift in [2Fe–2S]^2+^ clusters is only due to coupling of the nuclear spin with the electron spin excited levels, which can be significantly populated at room temperature. These proteins feature a broad signal, usually unresolved, in the 40–30 ppm range and individual βCH_2_ protons from Cys bound to the two ions cannot be identified nor sequence specifically assigned (Fig. 1c) [15, 23].

Upon reduction, the added electron can be localized on a single iron ion, and therefore, the iron pair is described as a Fe^3+^–Fe^2+^ pair, or the extra electron can be delocalized over the cluster [19, 24]. For localized valence cases, as observed in plant-type ferredoxins, when antiferromagnetic coupling occurs, the electron spin relaxation rates of both iron ions increase, and therefore, the coupled nuclear spins relax slower. As a consequence, NMR signals become sharper than those in the oxidized [2Fe–2S]^2+^ form, in particular for βCH_2_ bound to the purely Fe^2+^ ion (Fig. 1d). According to the theoretical model developed for two magnetically coupled metal ions [15, 20], the isotropic shifts decrease with increasing temperature (Curie-type behavior) for cysteines bound to the Fe^3+^ ion and increase with increasing temperature (anti Curie-type behavior) for cysteines bound to the Fe^2+^ ion. Therefore, the fitting of the experimental temperature dependence can provide a direct measure of the magnetic exchange coupling constant *J* [15]. The sequence-specific assignment of Cys residues bound to the cluster is the crucial step for the identification of the oxidation states of individual iron ions. Pioneering NOE experiments elegantly showed that, in [2Fe–2S]^+^ ferredoxins from plants and *algae*, the more reducible iron ion of the pair is that closer to the protein surface [25]. The Cys ligands bound to the more reducible ion form a larger number of hydrogen bonds than those bound to the other iron ion, in agreement with previous proposals [26]. This valence-localized model holds also in the case of Rieske ferredoxins [27].

In the case of ion pairs with delocalized valence, as observed in mammalian ferredoxins, the iron ions have much slower electron spin relaxation rates than in the localized valence pairs [28]. The pattern of chemical shifts can still be described with the model successfully used to account for the NMR properties of valence localized [2Fe–2S]^+^ ferredoxins, but nuclear relaxation is much faster thus determining much broader lines often undetectable for ^1^H signals (Fig. 1e). Sequence-specific assignment of metal binding residues is only possible via a combination of ^13^C, ^15^N and ^2^H experiments [23].

**Fig. 2:**
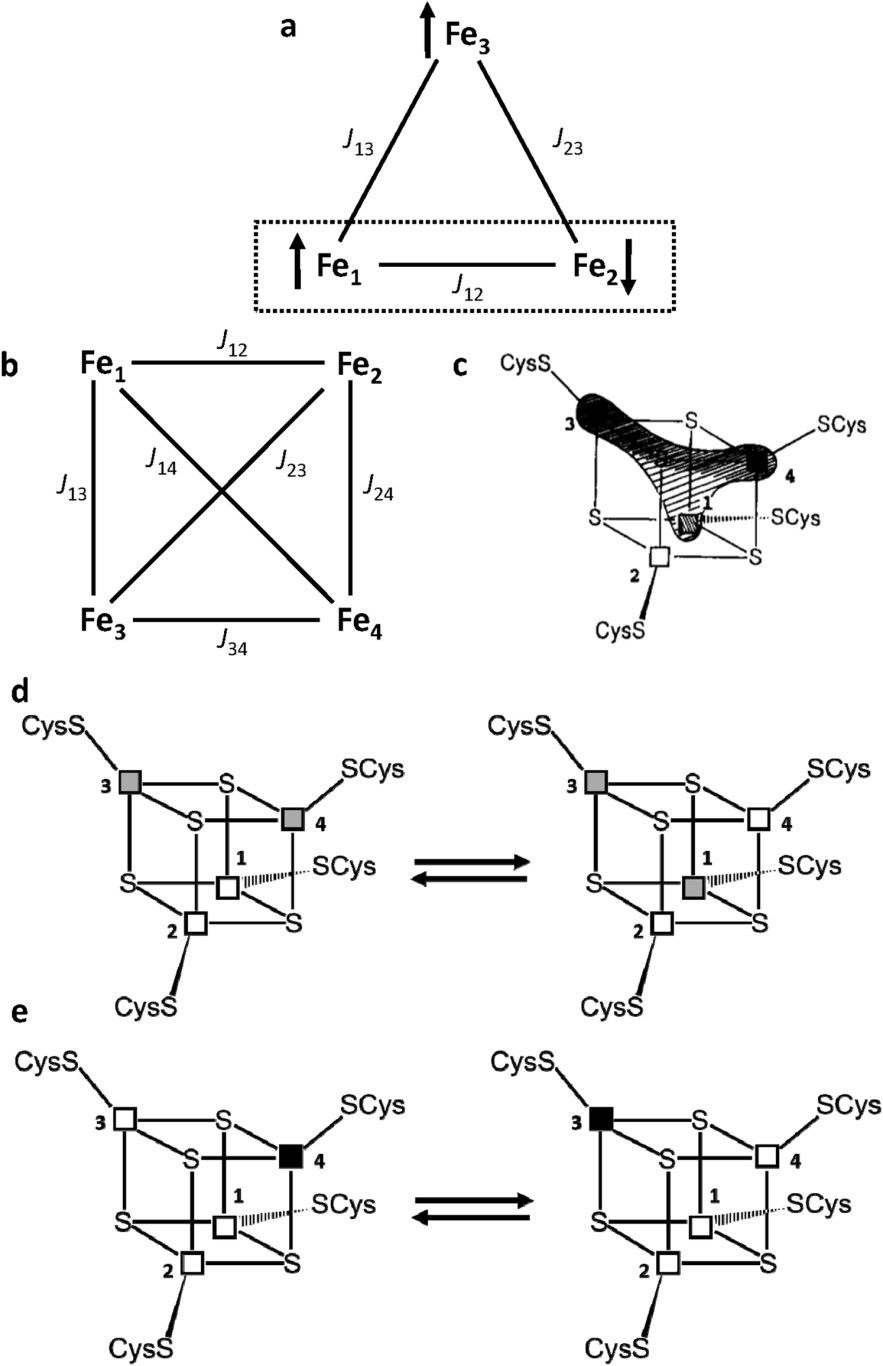
Magnetic coupling and electronic distribution in [3Fe–4S] and [4Fe–4S] clusters. Schematic representation of the spin frustration in a [3Fe–4S] cluster (**a**) and of the coupling scheme in a [4Fe–4S] cluster (**b**). **c**–**e** Electronic distribution in the [4Fe–4S]^3+^ clusters of HiPIPs: **c** the extra electron can be unevenly distributed among the iron ions Fe_1_, Fe_3_ and Fe_4_; **d** a chemical equilibrium between two different electronic distributions in the cluster, where mixed-valence Fe^2.5+^–Fe^2.5+^ iron ion pairs are represented as grey squares and purely Fe^3+^–Fe^3+^ pairs are represented as white squares; **e** illustration of the resonance between two limit formulas. Fe^3+^ and Fe^2+^ ions are represented as white and black squares, respectively

Discovered about 10 years later than the other Fe–S clusters [29], the [3Fe–4S] clusters are available in two oxidation states as well but, at variance with the [2Fe–2S] case, have paramagnetic ground states. The oxidized form, [3Fe–4S]^+^, contains three high spin Fe^3+^ ions. A total ground electron spin *S* = 1/2 level is observed, arising from slight inequivalence among the three *J*_*ij*_ values and from spin frustration [30, 31]. As shown in Fig. 2a, when *J*_12_ > *J*_13_ = *J*_23_, Fe_1_ and Fe_2_ form an antiferromagnetically coupled pair; as a consequence, Fe_3_ cannot be antiferromagnetically coupled to both Fe_1_ and Fe_2_ and remains with *S* = 5/2, while the Fe_2_–Fe_3_ iron pair has a subspin *S* = 2. Observed hyperfine shifts are in the 40–0 ppm range (Fig. 1f) and therefore, they are similar to the situation observed in [2Fe–2S]^2+^ case; however, nuclear relaxation is much slower and NMR signals are relatively sharp and easy to be identified [32]. Indeed, temperature dependence of the βCH_2_ protons signals of the iron bound cysteines is similar to the situation of [2Fe–2S]^2+^ case, i.e., signals from cysteines bound to the “frustrated” Fe_3_ ion have a Curie temperature dependence, while those arising from the Fe_1_–Fe_2_ pair (*S* = 2) have an opposite behavior with temperature. Therefore, sequence-specific assignment and iron identification within the scheme of Fig. 2a was achieved and contributed significantly to the understanding of the magnetic coupling scheme in [3Fe–4S] clusters [33–35]. In the reduced [3Fe–4S]^0^ state, the extra electron is delocalized on a ferromagnetically coupled iron pair [36]. The total *S* = 2 ground state [37] is such that NMR signals from cluster bound residues are too broad and/or shifted too far to be detected.

In [4Fe–4S] clusters, there are three possible/available states, [4Fe–4S]^3+/2+/1+^. The magnetic coupling scheme increases in complexity, as six *J*_*ij*_ magnetic coupling constants are needed to describe the system (Fig. 2b). In the [4Fe–4S]^2+^ case, the situation is described by two, identical, valence delocalized, Fe^3+^–Fe^2+^ pairs, that are antiferromagnetically coupled each other [38]. The electron spin energy levels diagram has a diamagnetic *S* = 0 ground state and the cluster has four equivalent iron ions, formally Fe^2.5+^. As in the case of oxidized [2Fe–2S]^2+^, paramagnetism arises from excited states [5, 6]. Typical spectrum is depicted in Fig. 1g. The observed contact shifts are smaller than those in [2Fe–2S]^2+^ proteins, indicating that, at room temperature, the excited levels are less populated, and therefore, larger *J* values than in the [2Fe–2S]^2+^ case are operative [39, 40]. Compared to [2Fe–2S] ferredoxins, shorter electron spin relaxation times determine sharper signals for Cys βCH_2_/αCH protons, which made possible the sequence-specific, stereospecific assignment of all eight βCH_2_ signals of the iron bound cysteines [40–42]. It was also found that contact shifts of Cys βCH_2_ protons depend on the Fe–S–C–H dihedral angle [43]; this angular dependence was successfully converted into structural constraints within solution structure calculations (see later) [44].

The [4Fe–4S]^2+^ state can be either oxidized to [4Fe–4S]^3+^ or reduced to [4Fe–4S]^+^; in small electron transfer proteins, the number of hydrogens bonds with the sulfur atoms of the cluster is the driving force for stabilizing one of the possible oxidation state pairs [45, 46], while water and peptide dipoles [47], electrostatic energy [48] and aromatic residues around the cluster [49, 50] provide a fine tuning of the reduction potential.

Upon cluster reduction to [4Fe–4S]^+^, the ground state is paramagnetic. The electronic situation can be described by the combination of a mixed valence Fe^2.5+^–Fe^2.5+^ pair and by a purely ferrous pair, Fe^2+^–Fe^2+^, which are antiferromagnetically coupled with each other to give an *S* = 1/2 ground state [38, 51]. While nuclear relaxation rates are very similar to those observed in the [4Fe–4S]^2+^ case [52], the magnitudes of the observed contact shifts as well as their temperature-dependence are quite distinctive. Signals from Cys βCH_2_/αCH protons are spread over a 65–5 ppm range (Fig. 1h). The relatively sharp linewidths of the signals made possible their sequence-specific assignment, while their temperature dependence allowed us to identify the oxidation state (i.e., Fe^2+^ or Fe^2.5+^) of each individual iron ion.

Individual oxidation states of iron ions in [4Fe–4S] clusters were identified for the first time in [4Fe–4S]^3+^ proteins, which represent the most favorable situation for solution NMR studies. In [4Fe–4S]^3+^ proteins, the electron spin relaxation times of the paramagnetic centers are shorter than those in the [4Fe–4S]^2+^ and [4Fe–4S]^+^ clusters, because of larger magnetic couplings among iron ions. Therefore, signals are sharper and easier to be sequence specifically assigned (Fig. 1i). Theoretical models involving double exchange contributions [18], spin frustration [53] and asymmetric model compounds [54] contributed to the understanding of the electronic structure of [4Fe–4S]^3+^ clusters in proteins, which have been described as a pair of two purely ferric (Fe^3+^–Fe^3+^) iron ions and a delocalized valence pair (formally Fe^2.5+^–Fe^2.5+^), which are antiferromagnetically coupled each other giving rise to a *S* = 1/2 ground state [55, 56], essentially having a magnetic coupling scheme analogous to the [4Fe–4S]^+^ case.

The favorable NMR properties of the [4Fe–4S]^3+^ cluster and the availability of a series of homologous proteins from different bacterial sources, characterized by high reduction potential values, spanning the + 450/+ 50 mV range (termed High Potential Iron Protein or HiPIPs) provided NMR spectroscopists with an exemplary case. Sequence-specific assignments of Cys residues bound to the cluster were performed, and the two iron ions constituting the mixed valence pair and those forming the purely ferric pair were identified [40–42, 57–60]. It was observed that the electronic distribution within the cluster varies from one protein to another, which can be described either with a “low symmetry distribution” in which the extra electron (considering that a [4Fe–4S]^3+^ cluster can be formally viewed as 4Fe^3+^ plus one electron) is unevenly distributed among the iron ions (Fig. 2c), or with a chemical equilibrium between two different electronic distributions within the cluster, the position of the equilibrium being determined by the electric field produced by the charges of the protein atoms around the cluster and by the metal ligands (Fig. 2d) [60]. The [4Fe–4S]^3+^ system can also be described by introducing a double exchange term [61], describing the resonance delocalization of the extra electron as depicted in Fig. 2e. It was found that, given the consensus sequence for cluster binding to HiPIPs, i.e., Cys_1_-X-X-Cys_2_-X_*n*_-Cys_3_-X_*m*_-Cys_4_, the iron ion bound to Cys_4_ (Fe_4_) is the most reducible iron ion and it is in a mixed valence state in all the investigated HiPIPs from different bacterial sources; Fe_1_ and Fe_3_ share the extra electron and interchange their character when passing from one protein to another, while Fe_2_ is the less reducible iron ion and has a purely ferric character throughout the series (Fig. 2c, d) [60].

## Solution structures of Fe–S proteins and beyond

The first solution structure of a paramagnetic protein determined by NMR was that of *E. halophila* HiPIP I, containing a reduced [4Fe–4S]^2+^ cluster [62]. This structure represented a breakthrough: until then, there was a common and somehow dogmatic belief that NMR structures of paramagnetic proteins were impossible to obtain, due to the lack of information in the proximity of the paramagnetic center. Indeed, scalar and dipolar connectivites are quenched by the presence of unpaired electron spins, but tailored 1D NOE, 2D TOCSY/NOESY experiments provide sufficient structural information to obtain low RMSD structures also around the Fe–S cluster. Furthermore, the hyperfine interaction is, per se, a source of additional constraints, which can be implemented within standard structure calculations programs to circumvent and possibly compensate the loss of structural information [63].

After the first NMR structure of reduced *E. halophila* HiPIP I, many NMR structures were determined for several Fe–S proteins in different oxidation states [44, 64–73]. For all of them, tailored approaches were used to compensate the quenching of the cross peaks arising from scalar and dipolar couplings, and to obtain structures with a low RMSD values in the proximity of the cluster. Recently, NMR structures of the holo form of some Fe–S proteins involved into the mitochondrial ISC assembly machinery [74] and of the NO sensing protein Wb1, containing a [4Fe–4S]^2+^ cluster, have been solved [75]. Structures of Fe–S proteins in their apo form or with a diamagnetic metal ion replacing the Fe–S cluster, determined with the standard/classic solution NMR approaches, are also available [76–82]. The latter approaches were also used to obtain solution NMR structures of holo proteins, but without structurally defining the area surrounding the cluster [83–85], i.e., no coordinates are given for the cluster atoms, and for the residues coordinating and/or belonging to the cluster environment. Hybrid approaches, where the absence of direct structural information in the proximity of the paramagnetic center was compensated by the use of homology models, have also been used [86–89].

The analysis of the structures of the same protein in two different oxidation states for several Fe–S proteins showed that differences in chemical shifts, unobserved residues, internal mobility and thermodynamic stability are suitable data to map subtle changes between the two different oxidation states [90–94]. On the contrary, no structural differences are observed in the solution structures at the available resolution. Even the solution structures of some HiPIPs, which are available for both oxidation states with a very high resolution, indicated that oxidation-state dependent structural rearrangements are too small to be observable [64], consistent with the low reorganization energy present in electron transfer proteins [95]. Redox-dependent structural differences were instead observed in [2Fe–2S] putidaredoxin, using a combination of diamagnetic restraints, paramagnetic restraints and residual dipolar couplings measured in orienting systems [96].

Chemical shift differences were widely explored to map transient interactions, as will be discussed in the next section. However, it should be always taken into account that, in paramagnetic proteins, chemical shift differences can arise from both structural changes, and changes of the hyperfine contributions (either a change of the hyperfine coupling constant or a change of the magnetic susceptibility anisotropy tensor). The two effects need to be disentangled for a proper analysis of the available NMR information. In the case of Fe–S proteins, the hyperfine contributions to the chemical shifts are limited to Fe–S cluster-bound ligands and to those residues that are hydrogen-bonded to the cluster. Hyperfine shifts are often more immediate and sensitive than NMR structures to monitor chemical events. For example, the transition from a native state to high energy species in an unfolding process, and the way how the folding/unfolding process is triggered by the electron transfer in electron-transfer proteins, can be followed by simple 1D ^1^H NMR experiments [97]. In the case of two [4Fe–4S] clusters containing ferredoxins, it was possible to measure the inter-cluster electron self-exchange rates and compare them with the exchange rates observed between oxidized, partly reduced, and fully reduced states [43]. Hyperfine shifts on ^15^N nuclei were also used to monitor the hydrogen-bonding network of residues surrounding the cluster [27, 98].

Many experimental approaches have been developed to collect structural information on paramagnetic proteins, and many of them turned out to be suitable for Fe–S proteins: dihedral angle constraints from Karplus-type equations, derived by considering the through-bond unpaired electron spin density delocalization onto the ligands [43], *T*_1_ and *T*_2_ relaxation-based constraints [68, 99], and ^13^C direct detection-based approaches [100–103] were successfully applied to Fe–S proteins. The magnetic anisotropy of the Fe^3+/2+^ ions in a tetrahedral environment is quite low, thus limiting to a few cases the use of paramagnetism-induced residual dipolar couplings and pseudocontact shifts as a source of structural information [104, 105], while cross-correlation phenomena [106] are not, at least so far, suitable for exploitation in Fe–S proteins.

**Fig. 3:**
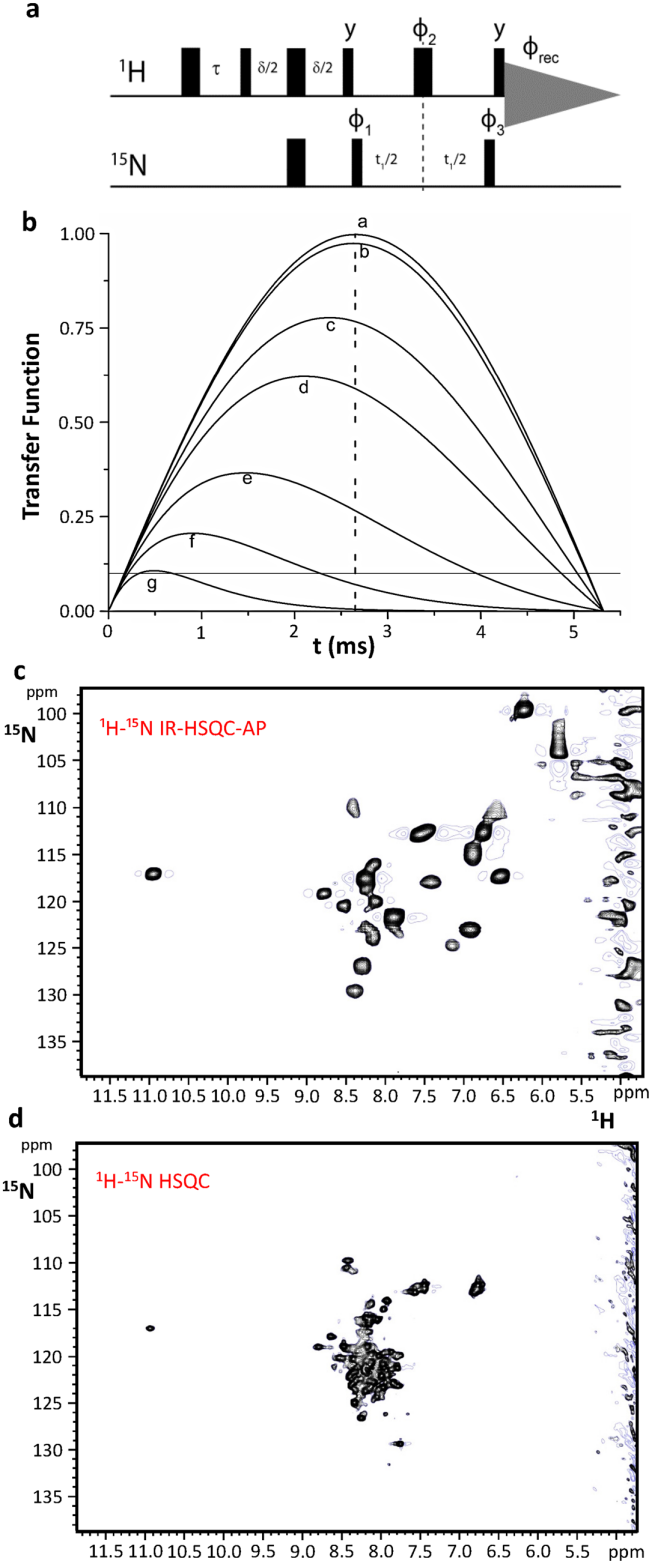
The ^1^H–^15^N IR-HSQC-AP NMR experiment: a new tool for paramagnetic Fe–S proteins. Schematic drawings of the pulse sequence of the ^1^H–^15^N IR-HSQC-AP NMR experiment. The inversion recovery delay *τ* and the coherence transfer delay *δ* must be chosen according to, respectively, *T*_1_ and *T*_2_ relaxation properties of the signals of interest. **b** Efficiency of an INEPT transfer function at different ^1^H *T*_2_ values: *b* 100 ms, *c* 10 ms, *d* 5 ms, *e* 2 ms, *f* 1 ms, *g* 0.5 ms. Relaxation is neglected in *a*. Letters have been drawn at the correspondence of the maximum values for each transfer function. A dashed line is shown at the 2.65 ms of INEPT step (94 Hz for ^1^H–^15^N *J* coupling). A solid line is shown in correspondence of 10% transfer efficiency. The latter is a limit threshold below which direct excitation of ^15^N spins should replace the INEPT step in the first part of the sequence. **c** Optimized ^1^H–^15^N IR-HSQC-AP experiment vs. **d** standard ^1^H–^15^N HSQC experiment acquired on 500 MHz at 298 K on the [2Fe–2S]^2+^-CIAPIN1 domain of human anamorsin

In more recent years, the use of NMR allowed the characterization of various aspects of Fe–S proteins in the studies of the complex machineries responsible for the biogenesis of Fe–S proteins. Structural properties, recognition patterns characterized by weak transient protein–protein interactions, and transient metal binding sites can be successfully addressed by NMR [107]. Small electron transfer proteins extensively studied in the previous decades have been used as model systems for more complex cases, in which conformational flexibility and protein–protein interactions make the investigation more challenging. The combination of 2D HSQC tailored to paramagnetic systems and inversion recovery (IR) lead to the development of IR-HSQC-antiphase (AP) [108, 109], a 2D experiment designed to provide both additional assignment and relaxation-based structural information for those cases in which information from contact shifts alone cannot be obtained. The use of this pulse sequence (Fig. 3a) turned out to be particularly helpful for systems such as [2Fe–2S]^2+^ proteins, characterized by efficient paramagnetic relaxation and by the absence of hyperfine shifts for all residues other than cluster ligands. The inversion recovery delay *τ* selects the signals according to *T*_1_ relaxation, while a customized choice of the coherence transfer delay *δ* allows us to optimize it according to *T*_2_ relaxation properties (Fig. 3b). Therefore, many signals affected by paramagnetic relaxation are buried under the bulk diamagnetic envelope. In the case of the CIAPIN1 domain of human protein anamorsin, the [2Fe–2S]^2+^ clusters are expected to induce minimal hyperfine shift and sizable paramagnetic nuclear relaxation on ^1^H spins that do not belong to metal coordinating cysteine residues, but are within a ~ 10 Å distance from each of the two iron ions. Consistent with these expectations, the ^1^H–^15^N-HSQC spectrum of the protein, recorded under standard conditions (Fig. 3d), shows only 71 out of 108 expected backbone NH signals. About 30% of the resonances remain unobserved due to paramagnetic broadening or exchange contributions. As shown in Fig. 3c, 10 additional HN signals, completely absent in standard NMR experiments, are present in the ^1^H–^15^N IR-HSQC-AP experiment. Furthermore, three backbone HN signals, barely detectable in standard experiments, significantly increase their intensity. Acquisition of spectra with different IR delays and the analysis of integrated intensity of the ^1^H–^15^N resonances allowed us to measure the *T*_1_ values for 12 out of 13 ^1^H signals. This has provided precious information on the relative positions of related residues with respect to the [2Fe–2S] cluster, which has been used to obtain a structural model of the CIAPIN1 domain of anamorsin [99].

## The contribution of solution NMR to understand molecular aspects of Fe–S cluster trafficking and assembly in humans

**Fig. 4:**
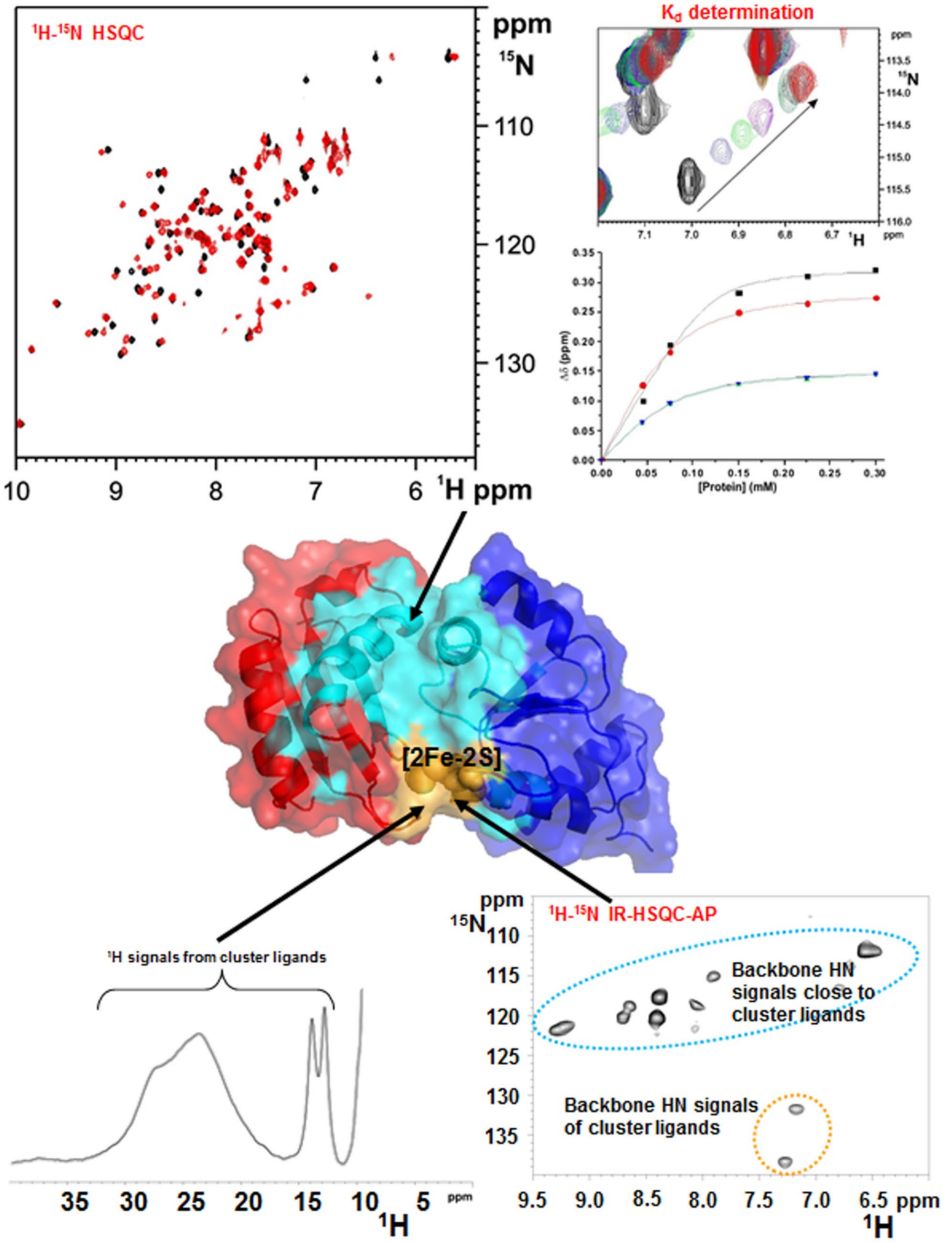
Solution NMR as a tool to investigate weak, transient protein–protein interactions in Fe–S protein maturation pathways. Weak and transient protein–protein interactions are detected by following backbone NH chemical shift changes occurring in a standard ^1^H–^15^N HSQC NMR experiment and in a ^1^H–^15^N IR-HSQC-AP NMR experiment, upon titrating ^15^N-labeled protein with the unlabeled protein partner and vice versa. Standard ^1^H–^15^N HSQC experiments allow the identification of protein–protein interacting regions far from the paramagnetic Fe–S cluster (showed in cyano), and to estimate the dissociation constant (*K*_d_) of the observed interaction. ^1^H–^15^N IR-HSQC-AP NMR experiment allows to identify protein–protein interacting regions close to the paramagnetic Fe–S cluster (showed in yellow). 1D ^1^H NMR experiment provides information on the kind of Fe–S cluster(s) bound or assembled on a target protein or protein–protein complex, on the redox state(s) of the cluster(s), and on the cluster ligands

The main advantage of solution NMR in the characterization of cellular pathways involving numerous interacting proteins, consists in the possibility of investigating, at the atomic level, weak transient protein–protein interactions, which are de facto difficult to be characterized at high resolution by other methodologies [110, 111]. The cellular pathways responsible for the maturation iron–sulfur proteins in humans are sequential, multistep processes, overall comprising at least 30 interacting proteins involved in two distinct machineries: the mitochondrial Fe–S cluster (ISC) assembly machinery, and the cytosolic Fe–S protein assembly (CIA) machinery, respectively composed of 17 and 13 proteins [112–114]. By combining standard and tailored solution NMR experiments, it was possible to describe structural and mechanistic aspects of the Fe–S cluster transfer and assembly processes occurring in these two machineries, which involve the formation of transient complexes. 1D ^1^H NMR experiments optimized to study the proximity of paramagnetic centers can provide information on the type of Fe–S cluster(s) bound or assembled on a target protein or in a protein–protein complex, as well as on the redox state(s) of the cluster(s); standard ^1^H–^15^N HSQC and ^1^H–^15^N IR-HSQC-AP NMR experiments can detect weak and transient protein–protein interactions through NH chemical shift mapping upon titration of one ^15^N-labeled partner (apo or holo) with the other unlabeled protein (apo or holo) (Fig. 4). These NMR data allowed us to identify the protein–protein interface on the interacting proteins, to have a good estimate of their affinity, of the stoichiometry of the interaction, and of the binding specificity (Fig. 4). It was also possible to obtain a structural model of protein complexes that bind a Fe–S cluster, via an experimentally driven docking approach (exploiting the HADDOCK program [115]), by integrating NMR chemical shift perturbation analysis with other experimental data derived from EPR, NMR tailored to paramagnetic systems and mutagenesis [116].

For several years, solution NMR has been extensively exploited to investigate molecular aspects of the ISC machinery of bacteria [117, 118]. In particular, *E. coli* has emerged as the model organism providing the greatest insights into the mechanistic details for Fe–S cluster biosynthesis and delivery to target proteins in the ISC machinery. In those studies, solution NMR data have been quite often integrated with other techniques, such as small-angle X-ray scattering (SAXS), optical spectroscopies, Mössbauer, X-ray crystallography and molecular dynamic simulations [119, 120]. An NMR-based integrated approach has been also applied to the structural characterization of isolated human proteins of the ISC and CIA assembly machineries [121–125], and, more recently, especially by our group, to investigate interaction networks involving proteins of the human ISC assembly and CIA machineries [99, 126–132]. In the following sections, we present how solution NMR studies contributed to describing protein–protein interactions in both human ISC and CIA assembly machineries.

### Solution NMR spectroscopy in the human ISC assembly machinery

The human proteins of the ISC assembly machinery are all soluble proteins located in the mitochondrial matrix. In the current working model, a [2Fe–2S] cluster is de novo synthesized on the scaffold protein ISCU2 by a high molecular weight complex (HMW complex hereafter), composed of five proteins (ISCU2, NFS1, frataxin, ISD11 and the acyl carrier protein) [133–135]. On the basis of yeast in vivo data [136–138], it has been proposed that the subsequent step in the human ISC assembly process consists of the transfer of the newly synthetized cluster to the mitochondrial monothiol glutaredoxin, GLRX5, which acts as a Fe–S cluster transfer protein, inserting the cluster into mitochondrial [2Fe–2S] protein targets. GLRX5 can also transfer the [2Fe–2S] cluster to the protein complex acting late in the ISC assembly machinery for generating [4Fe–4S] clusters [139]. The assembly of the [4Fe–4S] cluster is accomplished by two homologous proteins (ISCA1 and ISCA2), which contain three conserved cysteine residues in a CX_*n*_CGC sequence motif, and by a third protein (IBA57), whose function in the process is still unknown. It has been shown that these three proteins are strictly required for the maturation of mitochondrial [4Fe–4S] proteins, but not necessary for the maturation of mitochondrial [2Fe–2S] proteins in eukaryotes [140–144]. Once assembled by ISCAs, the [4Fe–4S] cluster is inserted into mitochondrial [4Fe–4S] protein targets, being this process often dependent on other ISC accessory proteins, such as NFU1, BOLA3 and NUBPL [81, 145–147].

In the de novo formation of a [2Fe–2S] cluster, the majority of the proteins of the human ISC assembly machinery forms permanent interactions featuring tight binding affinities [133–135, 148]. The human HMW complex assembling the [2Fe–2S] cluster has been, indeed, isolated as a stable unit from *E. coli* cells. Due to its very high molecular mass, X-ray crystallography and cryo-EM are the most appropriate techniques to structurally characterize the complex at the atomic level and have, indeed, successfully generated structural models of the complex or sub-complex forms [133–135]. In this first step of the human ISC machinery, solution NMR was crucial to investigate protein–protein interactions that occur between the HMW complex and mitochondrial [2Fe–2S] ferredoxins. Through these interactions, electrons are provided to the HMW complex by a mitochondrial [2Fe–2S] ferredoxin for generating the [2Fe–2S] cluster on ISCU2 [74, 132, 149, 150]. As it often occurs in protein–protein interactions driving electron transfer [151, 152], the interaction between the HMW complex and [2Fe–2S] ferredoxins is transient and with a µM range affinity; therefore, solution NMR was the successful approach to characterize the process at the molecular level [74, 132]. In such studies, it was shown that the regions of ferredoxins recognizing the multi-component complex are close to the [2Fe–2S] cluster, and that no interaction occurs between apo forms of ferredoxins and the HMW complex.

Solution NMR largely contributed to investigate the subsequent steps of the ISC assembly machinery, being able to provide a detailed model of how the [2Fe–2S] clusters, de novo synthetized in the multi-component complex, couple with each other to form a [4Fe–4S] cluster. This process involves four interacting proteins as mentioned above, i.e., GLRX5, ISCA1, ISCA2 and IBA57.

**Fig. 5:**
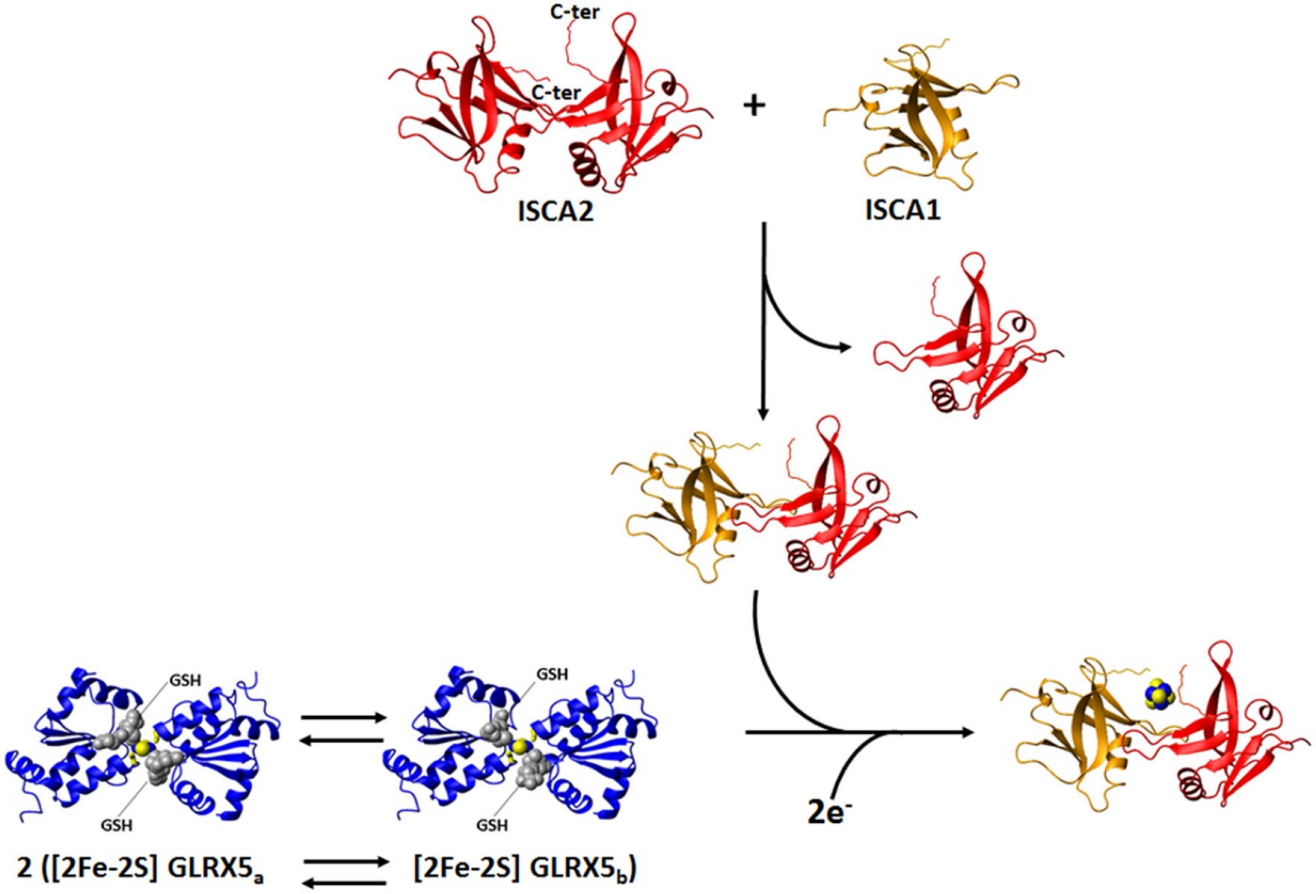
The NMR contribution to the investigation of [4Fe–4S] clusters formation in the mitochondrial iron–sulfur cluster assembly machinery. On the basis of standard and paramagnetic systems-tailored NMR experiments, we provide a model for the transfer of two [2Fe–2S]^2+^ clusters from GLRX5 to ISCA1–ISCA2 hetero-dimeric complex: in solution dimeric [2Fe–2S]^2+^ GLRX5 has two states in equilibrium with each other, differing in the binding mode of the GSH molecules ([2Fe–2S] GLRX5_a_ and [2Fe–2S] GLRX5_b_); dimeric [2Fe–2S]^2+^ GLRX5 specifically transfers the cluster to apo ISCA1–ISCA2 hetero-dimeric complex via an associative process that involves a transient protein–protein intermediate; [2Fe–2S] GLRX5_b_ is more reactive than [2Fe–2S] GLRX5_a_ to donate the cluster; ISCA1–ISCA2 hetero-dimeric complex is obtained in solution by exchanging one subunit of the ISCA2 symmetric dimer with one subunit of ISCA1, which, as isolated protein, is present in solution in a monomer–dimer equilibrium; the two [2Fe–2S]^2+^ clusters received preferentially by [2Fe–2S] GLRX5_b_ are reductively coupled on ISCA1–ISCA2 hetero-dimeric complex to form a [4Fe–4S]^2+^ cluster

The crystal structure of [2Fe–2S] GLRX5 shows a homotetrameric structural organization (a dimer of dimers), where two [2Fe–2S] clusters are coordinated by four protein subunits and four GSH molecules and buried in the tetramer [153]. In such conformation, the two clusters are not easily accessible by cluster receiving apo proteins, a circumstance that impairs any possible cluster transfer process. This structural organization would make the proposed chaperone function of GLRX5 difficult to occur. However, it was shown by NMR that apo GLRX5 is monomeric in solution, and that it undergoes dimerization only upon cluster binding [128]. These data indicate that the tetrameric state observed in the crystal structure of [2Fe–2S] GLRX5 is likely determined by crystallization conditions, thus making the tetrameric state of [2Fe–2S] GLRX5 of poor functional relevance to the cluster transfer process. The combination of standard ^1^H–^15^N HSQC and ^1^H–^15^N IR-HSQC-AP NMR experiments allowed the identification of the residues affected by [2Fe–2S] cluster binding [128]. By mapping the chemical shift variations between apo and [2Fe–2S] GLRX5 on the crystallographic structure of [2Fe–2S] GLRX5 [153], we found that the regions affected by cluster binding are in a 10-Å radius sphere centered on the [2Fe–2S] cluster, which bridges the two subunits of the dimer. Backbone NH signals of 11 residues located inside this sphere were not detected in the standard ^1^H–^15^N HSQC experiment, but 9 of them were recovered through the ^1^H–^15^N IR-HSQC-AP NMR experiment [109]. Their ^1^H *T*_1_ values increase with increasing distance from the cluster with the expected *r*^−6^ dependence. Overall, the NMR data indicate that the dimeric state of [2Fe–2S] GLRX5 in solution adopts essentially the same structural arrangement as observed for the dimer in the crystal structure. However, ^1^H–^15^N IR-HSQC-AP and 1D ^13^C NMR experiments tailored to paramagnetic systems also showed that dimeric [2Fe–2S] GLRX5 exists in solution as a mixture of two species in equilibrium each other (GLRX5_a_ and GLRX5_b_), as two sets of signals for the Fe–S ligand Cys 67, and for Ser 70 were identified [128]. Also in standard ^1^H–^15^N HSQC experiments, the six residues surrounding the “paramagnetic sphere” have two sets of NH signals, both sets having chemical shifts different from those of the apo protein. By mapping the residues experiencing two sets of signals on the dimeric structure of [2Fe–2S] GLRX5, we observed that they are all around the iron-bound GSH molecule. Among these eight residues, the two charged ones, Lys 101 and Asp 123, are particularly important to establish electrostatic interactions with the glycine carboxylate and the glutamate amine groups of GSH, respectively. All the available NMR data indicate that two dimeric species of [2Fe–2S] GLRX5 exist in solution, differing in the binding mode of the GSH molecule (Fig. 5). Apparently, in the crystal structure, [2Fe–2S] GLRX5 adopted one of the two forms. The possible functional relevance of the presence of these two species in equilibrium has then been addressed by characterizing the interaction between [2Fe–2S] GLRX5 and its protein partners ISCAs.

In all available bacterial apo and holo structures, ISCA proteins are either dimeric or tetrameric, and show different symmetries and different cluster ligands [154–157]. This conformational variability observed in bacterial ISCAs suggests that it is not very appropriate to transfer the structural and cluster coordination information acquired on bacterial proteins to human ISCAs. A detailed structural characterization of the human ISCAs is thus required before proceeding with protein–protein interaction studies with their partner proteins. In solution, apo ISCA2 is a symmetric dimer, with a well-structured α–β domain encompassing residues 50–140, and a completely unstructured C-terminus of 15 residues (Fig. 5) [82]. Backbone ^15^N NMR relaxation data showed a global rigidity of the α–β domain, but also identified a certain degree of backbone flexibility in the loop containing the Fe–S ligand Cys 79, located in the first position of the conserved CX_*n*_CGC motif. Also, the C-terminal tail of ISCA2, encompassing residues 141–154, and containing the other two conserved Fe–S binding Cys residues (Cys 144 and Cys 146), was found to be highly flexible. Therefore, NMR analysis indicated that all the regions containing the conserved Cys residues can easily undergo structural rearrangements to bind the cluster. Severe line broadening of a few backbone NH signals observed in the ^1^H–^15^N HSQC map of apo ISCA2 indicates that dynamic processes also occur at the dimer interface, suggesting an intrinsic propensity for the subunits to be swapped with other protein partners. This is, indeed, what occurs by mixing apo ISCA2 with apo ISCA1. This process has been characterized by solution NMR, performing ^1^H–^15^N HSQC NMR titrations of ^15^N-labeled apo ISCA1 or ISCA2, with, respectively, unlabeled apo ISCA2 or ISCA1 [82]. The two proteins form in solution a stable heterodimeric complex by exchanging, slowly on the NMR time scale, one subunit of the ISCA2 dimer with one subunit of ISCA1, which, as isolated apo protein, is present in solution as a monomer–dimer equilibrium (Fig. 5). A well-defined surface of interaction, which involves the subunit–subunit interface of homo-dimeric apo ISCA2, was also identified. The NMR data provided a clear evidence that a thermodynamically favored heterodimeric adduct between ISCA2 and ISCA1 is formed at the expense of the homodimeric species (Fig. 5). This is in agreement with in vivo data showing a tight interaction between ISCA1 and ISCA2 [158].

The 1D ^1^H spectrum of holo human ISCA2 as purified from *E. coli* cells showed a set of broad signals characteristic of [2Fe–2S]^2+^ cluster binding [82]. In contrast, upon chemical reconstitution, the 1D ^1^H spectrum is consistent with the presence of a mixture of [4Fe–4S]^+^ cluster-bound dimeric species and a minor [2Fe–2S]^2+^ cluster-bound species [82]. Backbone ^15^N relaxation data indicated that [2Fe–2S] or [4Fe–4S] cluster binding does not alter the quaternary structure of ISCA2 [82], at variance with what observed in the bacterial homologues [154–157]. Since each subunit of the ISCA2 dimer has three potential Fe–S cluster ligands (i.e., the conserved Cys 79, Cys 144, Cys 146), 1D ^1^H NMR experiments have been perfomed to identify the two pairs of [2Fe–2S] cluster ligands. The [2Fe–2S] cluster binding properties of Cys-to-Ser single mutants for each conserved cysteine were compared with those of the wild-type protein and their ^1^H NMR spectra analyzed [129]. From this study, it emerged that ISCA2 coordinates the oxidized [2Fe–2S]^2+^ cluster with two Cys 79, provided by each of the two subunits of the ISCA2 homodimer, with Cys 146 from one subunit of the homodimer and with Cys 144 from the other subunit of the homodimer. NMR data acquired on (^13^C, ^15^N) Cys selectively labeled wild-type ISCA2 protein suggested that this cluster coordination is also conserved once ISCA2 binds a [4Fe–4S] cluster [129].

Cluster transfer between [2Fe–2S] GLRX5 and the apo form of ISCA1 and ISCA2 was then characterized by performing NMR titrations. The NMR data indicated that cluster transfer occurs unidirectionally from GLRX5 to apo ISCA1 and ISCA2 and that the [2Fe–2S] GLRX5_b_ form is preferentially reacting relative to [2Fe–2S] GLRX5_a_ [128]. ^15^N NMR relaxation data showed that ISCA1 and ISCA2 receive the [2Fe–2S] cluster from [2Fe–2S] GLRX5 in their dimeric state. NMR data also showed that cluster transfer occurs via the formation of a low-populated protein–protein complex, with an interacting surface involving the GLRX5 region surrounding Cys 67 [128]. Finally, apo GLRX5 does not interact with apo ISCA1 or ISCA2, as no significant spectral changes are observed when the apo proteins are mixed together, indicating that the [2Fe–2S] cluster is essential for the formation of a weakly interacting protein–protein adduct. In conclusion, a model for the transfer of the [2Fe–2S] cluster from GLRX5 to ISCA1 and ISCA2 can be proposed on the basis of the NMR data: (1) dimeric [2Fe–2S] GLRX5 has two states in equilibrium with each other, [2Fe–2S] GLRX5_a_ and [2Fe–2S] GLRX5_b_; (2) dimeric [2Fe–2S] GLRX5 specifically transfers the [2Fe–2S]^2+^ cluster to apo ISCA1 and apo ISCA2 via an associative process that involves a weak transient protein–protein intermediate; and (3) [2Fe–2S] GLRX5_b_ is more reactive than [2Fe–2S] GLRX5_a_ to donate the cluster to apo ISCA1 and apo ISCA2. In conclusion, the equilibrium between [2Fe–2S] GLRX5_a_ and [2Fe–2S] GLRX5_b_ species is the trigging factor specifically driving cluster transfer to ISCAs proteins, and thus plays a functional role in the cluster transfer mechanism (Fig. 5).

Solution NMR was also applied to the investigation of [2Fe–2S] cluster transfer from GLRX5 to the heterodimeric ISCA1–ISCA2 complex. NMR data showed that two [2Fe–2S]^2+^ clusters are transferred from [2Fe–2S]^2+^ GLRX5 to the ISCA1–ISCA2 heterodimeric complex, and that the transfer occurs via the formation of a low-populated, transient protein–protein complex [82]. This supports a general cluster transfer mechanism occurring via an associative process between GLRX5 and either homodimeric ISCA1 or homodimeric ISCA2 or the heterodimeric ISCA1–ISCA2 complex. By monitoring NMR chemical shift changes on ^15^N-labeled ISCA1–ISCA2 upon interaction with [2Fe–2S] GLRX5, it was found that the two [2Fe–2S]^2+^ clusters received by GLRX5 were reductively coupled to form a [4Fe–4S]^2+^ cluster (Fig. 5). The same final [4Fe–4S]^2+^ species was obtained after transferring one [2Fe–2S]^2+^ from GLRX5 to apo ISCA2 homo-dimer, and then adding [2Fe–2S]^2+^ ISCA1 homo-dimer to the mixture. Standard ^1^H–^15^N HSQC and 1D ^1^H NMR experiments also provided detailed information on the mechanism of the formation of the [4Fe–4S]^2+^ cluster in the ISCA1–ISCA2 hetero-dimeric complex. By monitoring cluster transfer from [2Fe–2S]^2+^ GLRX5 to Cys-to-Ser single mutants of each conserved cysteine of ISCA2 (i.e., C79S, C144S, and C146S mutants), we were able to define the different roles of the cysteines in the cluster transfer process [129]. The NMR data supported a model in which the two C-terminal cysteines, located in the unstructured and flexible C-terminal tail of the ISCAs proteins, extract the [2Fe–2S]^2+^ cluster from GLRX5 by forming a transient, low-populated, cluster-mediated GLRX5-ISCAs intermediate, where the two cluster-binding GSH molecules of GLRX5 are substituted by the ISCA cysteines. This cluster-extraction mechanism from GLRX5 results in the formation of an ISCA1–ISCA2 species that binds the cluster via the four C-terminal cysteines. The latter species is, however, transient and no [2Fe–2S] cluster bound species can be isolated once Cys 79 is absent, i.e., upon chemical reconstitution of the C79S ISCA2 mutant [129]. Cys 79 is, however, not involved in the cluster transfer step, as the C79S ISCA2 mutant is still able to extract the [2Fe–2S]^2+^ cluster from GLRX5 [129], via the formation of the transient, low-populated intermediate with no cluster release in solution. However, the transfer is not as efficient as that observed for the wild-type protein. This suggests that cluster-binding affinity is lower once the cluster is coordinated via the C-terminal cysteines only (C79S ISCA2 mutant case) than once Cys 79 participates to cluster binding (wild-type ISCA2 case). According to this model, the C144S and C146S ISCA2 mutants, at variance with what happens in the C79S ISCA2 mutant, can be isolated upon chemical reconstitution [129]. Therefore, we propose that the species coordinating the cluster with the four C-terminal cysteines can evolve into a more thermodynamically favored species, which binds the [2Fe–2S]^2+^ cluster in the ISCA1–ISCA2 heterodimer by Cys 79 and Cys 144 of ISCA2, and Cys 57 and Cys 123 of ISCA1. 1D ^1^H NMR data showed, indeed, that this is the preferential coordination mode in wild-type ISCA2 for binding either [2Fe–2S] or [4Fe–4S] clusters, and presumably also in the heterodimeric ISCA1–ISCA2 complex. This mechanism would also make two of the C-terminal cysteines (Cys 146 of ISCA2 and Cys 121 of ISCA1) available for the coordination of a second cluster which can be extracted from GLRX5, upon the formation of another GLRX5-ISCA intermediate. This transient intermediate, which contains two [2Fe–2S]^2+^ clusters, might be the species that, by accepting two electrons from a still unknown physiological electron donor (Fig. 5), evolves to the final [4Fe–4S]^2+^ cluster-bound ISCA1–ISCA2 complex. A reductive coupling of two [2Fe–2S]^2+^ clusters, which is a general mechanism for generating a [4Fe–4S]^2+^ cluster [159, 160], would therefore, occur on the latter transient intermediate to form a [4Fe–4S]^2+^ cluster bound to the hetero-complex. The proposed molecular model agrees with in vivo data on yeast, which showed that all three conserved cysteines of Isa1 and Isa2, the yeast homologues of ISCA1 and ISCA2, are essential for the maturation of [4Fe–4S] proteins [161, 162].

### Probing the human CIA machinery with solution NMR spectroscopy

As it is for the mitochondrial ISC assembly machinery, the proteins of the human CIA machinery responsible for the synthesis, trafficking, and insertion of clusters into the cytosolic and nuclear Fe–S protein targets are all soluble. In cytoplasm, the ratio of human proteins containing [2Fe–2S] vs. [4Fe–4S] is 18:10 [163].

The current working model for cytosolic/nuclear [4Fe–4S] protein maturation envisages that a [4Fe–4S] cluster is assembled on a specific scaffold complex, formed by two cytosolic Fe–S cluster assembly factors, NUBP1 and NUBP2 [164–166]. The [4Fe–4S] cluster is then transferred to a high molecular weight complex composed by three proteins, named CIA targeting complex, that mediates its final incorporation into the cytosolic/nuclear targets [167, 168]. Other CIA accessory proteins are often required to assist in the incorporation of [4Fe–4S] clusters into specific protein targets [169, 170]. The origin of iron and sulfur ions used by the NUBP1 and NUBP2 scaffold complex to build the [4Fe–4S] cluster is still not identified. It has been proposed that cytosolic monothiol glutaredoxins work as cytosolic iron donors to cytosolic proteins and to Fe–S and to heme binding proteins [171–173]. This proposal was based on the fact that the cytosolic monothiol glutaredoxins play a role in intracellular iron trafficking and sensing, in iron homeostasis and hemoglobin maturation. Recently, it also emerged that cytosolic monothiol glutaredoxins can assist Fe–S protein maturation in the cytosol by acting as [2Fe–2S] cluster donors. The first work proposing a role of cytosolic monothiol glutaredoxins in cytosolic [2Fe–2S] cluster trafficking appeared two years ago, and it represents a very nice example of how in vitro solution NMR data predicted this function for the cytosolic monothiol glutaredoxins [127]. One year after that study, human monothiol glutaredoxin GLRX3 was shown, indeed, to work as a Fe–S cluster chaperone in human cells [174].

**Fig. 6:**
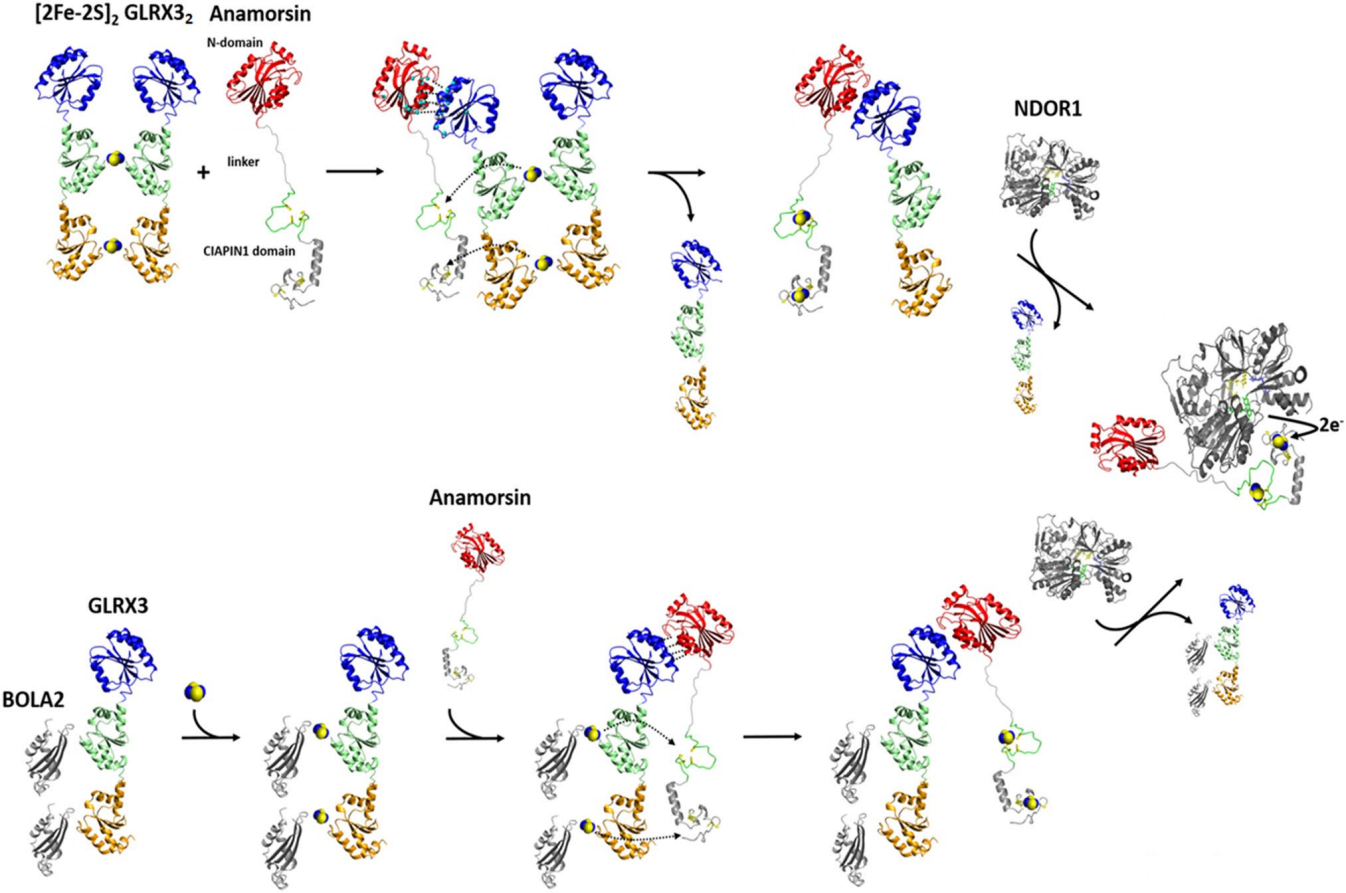
The NMR contribution to the elucidation of  the GLRX3-dependent anamorsin maturation pathway. Standard ^1^H–^15^N-HSQC and ^1^H–^15^N IR-HSQC-AP NMR experiments, combined with UV–Vis and EPR spectroscopy, showed that GLRX3 forms a 1:1 hetero-dimeric complex with anamorsin, in which both clusters from [2Fe–2S]_2_ GLRX3_2_ are transferred to anamorsin. In its mature holo state anamorsin interacts with NDOR1, forming a specific protein complex, where the anamorsin unstructured linker tightly interact with NDOR1, while the C-terminal CIAPIN1 domain of anamorsin, containing the [2Fe–2S] redox center, only transiently interacts, through complementary charged residues, with the FMN-binding domain of NDOR1 to perform the electron transfer reaction. Standard NMR experiments showed that the [2Fe–2S]_2_ GLRX3–BOLA2_2_ hetero-complex transfers in vitro both [2Fe–2S]^2+^ clusters to apo anamorsin, producing its mature holo state, and that this process goes via the same protein–protein recognition mechanism operating in the GLRX3-anamorsin interaction, i.e., specifically occurring between the N-terminal domains of the two proteins. The BOLA2–GLRX3 complex might be released in solution upon the interaction of holo anamorsin with NDOR1

The human proteome contains only one monothiol glutaredoxin in the cytosol, i.e., GLRX3, which consists of three domains: one N-terminal thioredoxin (Trx) domain with no Trx enzymatic activity, but functionally indispensable [171, 175], and two monothiol glutaredoxin (Grx) domains, each able to bind a glutathione-coordinated [2Fe–2S] cluster via protein dimerization (Fig. 6) [176, 177]. Yeast-two-hybrid and affinity capture-MS screens showed that in vivo GLRX3 binds anamorsin [178]. Anamorsin contains two domains: a N-terminal well-folded domain (N-domain, hereafter) of 172 residues and a largely unstructured C-terminal domain of 90 residues, named Cytokine-Induced Apoptosis INhibitor 1 (CIAPIN1, hereafter), essential for the viability of yeast [179, 180], and containing two highly conserved cysteine-rich motifs, easily able to independently bind a [2Fe–2S] cluster (Fig. 6) [99, 181]. These two domains are connected by a long flexible and unstructured linker of 50 residues [181] (Fig. 6).

Anamorsin is the appropriate protein partner to investigate the functional role of GLRX3 in transferring [2Fe–2S] clusters for these reasons: (1) it interacts with GLRX3 in vivo [178]; (2) it binds [2Fe–2S] and [4Fe–4S] clusters [121, 182, 183]; (3) the insertion of the Fe–S cluster into the yeast homologue of anamorsin, Dre2, depends on Grx3 and Grx4, the two cytosolic, functionally redundant yeast homologues of GLRX3 [171, 173]; (4) Fe–S cluster loading on Dre2 is independent of the cytosolic iron–sulfur protein assembly machinery [180].

The monomeric apo and dimeric [2Fe–2S]^2+^ cluster-bound forms of GLRX3 (hereafter apo GLRX3 and [2Fe–2S]_2_ GLRX3_2_, respectively) [184] were first characterized by NMR, showing that the Trx domain does not have intra- and inter-subunit interactions with the two Grx domains, nor with the Trx domain of the other monomer in [2Fe–2S]_2_ GLRX3_2_, being therefore, fully available to be potentially involved in protein–protein interactions [127]. This structural aspect prompted us to investigate whether the Trx domain of GLRX3 drives a specific protein–protein interaction with anamorsin. The analysis of NMR titration data, acquired after mixing the apo proteins, showed that apo-GLRX3 and apo-anamorsin form a 1:1 heterodimeric complex through their N-terminal domains and that the CIAPIN1 domain of anamorsin and the Grx domains of GLRX3 are not involved in any permanent interaction in this complex [127]. Molecular recognition between the N-terminal domains is, therefore, the crucial factor determining complex formation between the two proteins. A docking model of the complex, based on the NMR titration data, showed that the interaction occurs among the α-helical regions of the two domains, but also involves a negatively charged, Glu-rich, region (Glu 71, Glu 75, Glu 78, and Glu 81) of GLRX3 and a specific region of the unstructured linker of anamorsin, rich with positively charged Lys residues (Lys 175, Lys 180, Lys 181, Lys 187) [127]. These two regions stabilize protein–protein interactions through electrostatic recognition. Indeed, the exchange regime between the free and the bound proteins switched from fast/intermediate on the NMR time scale upon interaction of the N-terminal domains of GLRX3 and anamorsin, to slow when the N-domain of GLRX3 interacted with full-length anamorsin, in agreement with a significant increase in the protein–protein affinity when the linker is present. In conclusion, the NMR/biomolecular docking data defined, for the first time, the function of the N-domains of the two proteins and identified a role of the linker of anamorsin in stabilizing the protein–protein interaction [127].

Standard ^1^H–^15^N-HSQC and ^1^H–^15^N-IR-HSQC-AP NMR experiments, combined with UV–Vis and EPR spectroscopy, showed that [2Fe–2S]_2_ GLRX3_2_ forms a 1:1 hetero-dimeric complex with anamorsin, the same formed by the apo proteins, in which both clusters of GLRX3 have been unidirectionally transferred to the two cluster binding sites of the CIAPIN1 domain of anamorsin (Fig. 6) [127]. This means that the GLRX3 molecule in the 1:1 heterodimeric complex is in the apo state, that the N-terminal domains (Trx of GLRX3 and N-domain of anamorsin) interact in the complex, and that the C-terminal, cluster-binding domains (Grxs and CIAPIN1 domains) are not involved in a stable protein–protein interaction (Fig. 6). The interaction between the N-domains is a fundamental requisite in the cluster transfer mechanism to drive [2Fe–2S] cluster transfer from GLRX3 to the CIAPIN1 domain of anamorsin. We suggested that the protein–protein interaction between the N-terminal domains make the cluster binding domains, i.e., the Grx donors and the CAPIN1 acceptor, in the optimal reciprocal orientation for the cluster transfer to efficiently occur. Therefore, it appears that the transfer process from GLRX3 to anamorsin is a thermodynamically favored process under kinetic control. This mechanism guarantees that two [2Fe–2S] clusters are concomitantly transferred in a single molecular event to the target protein requiring two [2Fe–2S] clusters. This NMR-based study opens new perspectives on the cellular function of GLRX3 in humans, showing that GLRX3 function, by playing a key role in maturing anamorsin, is strictly linked to all anamorsin-dependent cellular processes. Anamorsin is a crucial early step component of the CIA machinery, being essential for the maturation of cytosolic/nuclear [4Fe–4S] proteins. Therefore, the decreased activities of the cytosolic [4Fe–4S] proteins IRP1 and GPAT observed by silencing human GLRX3 in HeLa cells [172] can be due to impairment of the GLRX3-dependent anamorsin maturation process. This makes, indeed, the CIA machinery unable to function, i.e., to assemble the [4Fe–4S] clusters of IRP1 and GPAT.

The matured holo form of anamorsin forms a stable complex with the cytosolic NADPH-dependent diflavin reductase NDOR1 in the cell [180]. This complex, which receives two electrons from the NADPH cofactor, has been proposed to act as a source of reducing equivalents for the assembly of target, but not to be a scaffold for Fe–S cytosolic proteins [99, 180]. This means that, once the cluster transfer from GLRX3 to anamorsin has occurred in the cell, the complex between the two proteins needs to be terminated, so that the functional process(es) performed by the mature form of anamorsin can proceed. Recently, we found that the N-terminal domain of anamorsin is not involved in protein–protein recognition, and that the C-terminal CIAPIN1 domain of anamorsin, containing the [2Fe–2S] redox center, only transiently interacts, through complementary charged residues, with the FMN-binding domain of NDOR1 to perform the electron transfer reaction [99]. On the contrary, the unstructured linker of anamorsin tightly interacts with NDOR1, inducing the formation of a specific and stable protein complex [99]. On this basis, we suggested that upon interaction of the GLRX3-anamorsin complex with NDOR1, the linker of anamorsin weakens its interaction with GLRX3, while favoring the interaction with NDOR1 (Fig. 6). As the stabilizing effect of the linker on GLRX3-anamorsin interaction is lost, the binding affinity of the N-terminal domain of GLRX3 with that of anamorsin is decreased, and, as a consequence, the complex between GLRX3 and anamorsin might switch to the complex between anamorsin and NDOR1. The linker interaction is thus able to modulate the formation and release of the various protein–protein complexes, enabling the redox-competent state of anamorsin to receive electrons from NDOR1. The high flexibility and intrinsic disorder of the linker fits well with the interaction with multiple partners, as commonly observed for intrinsically disordered proteins/regions.

Several lines of evidence, including affinity purification, yeast two-hybrid studies and gene co-occurrence analysis indicated that the monothiol Grxs functionally and physically interact with another widely conserved protein family, the BolA-like proteins [176, 185–187]. Eukaryotic organisms contain BolA-like proteins in both mitochondria and cytoplasm. In yeast, mitochondrial BolA1 and BolA3 proteins are involved in the ISC assembly machinery, working as specific mitochondrial ISC assembly factors that facilitate [4Fe–4S] cluster insertion into a subset of mitochondrial proteins, such as lipoate synthase and succinate dehydrogenase [81, 147]. On the contrary, cytosolic yeast BolA2 protein plays a role in iron homeostasis [188]. In humans, the functional role of the mitochondrial BOLA1 and BOLA3 proteins is still not clearly defined, but it was found that they form a hetero-dimeric complex with GLRX5 in both apo and [2Fe–2S]-cluster bound states [81]. NMR data, combined with other spectroscopic information, allowed us to obtain an experimentally driven docking model of [2Fe–2S] cluster-bridged dimeric BOLA1–GLRX5 and BOLA3–GLRX5 complexes, showing that the BOLA1–GLRX5 complex coordinates a reduced, Rieske-type [2Fe–2S]^+^ cluster, while an oxidized, ferredoxin-like [2Fe–2S]^2+^ cluster is present in the BOLA3–GLRX5 complex [116]. It also appeared that the [2Fe–2S] BOLA1–GLRX5 complex is preferentially formed over the [2Fe–2S] BOLA3–GLRX5 complex, as a result of a higher cluster binding affinity. The different structural and redox properties observed for the two [2Fe–2S] BOLAs–GRX5 complexes as well as their different stability suggested that they can have a diverse molecular function. Possibly, [2Fe–2S] BOLA1–GLRX5 complex might be involved in electron transfer processes, while the [2Fe–2S] BOLA3–GLRX5 might be involved in cluster transfer versus client proteins along the ISC assembly pathway. However, functional data are required to verify such proposed molecular function of the mitochondrial BOLAs–GLRX5 complexes. As it is for mitochondrial BOLAs and GLRX5 proteins, cytosolic BOLA2 and GLRX3 proteins form a hetero-complex in both the apo and the [2Fe–2S] cluster bound forms [177, 184]. In both cases, this hetero-complex is composed by two BOLA2 molecules and one GLRX3 molecule. Recently, solution NMR contributed to unravel the functional role of this complex. NMR titration data showed that apo BOLA2 interacts simultaneously with both Grx domains of GLRX3 with an apparent dissociation constant of 25 μM, without showing a preferential interaction toward one of the two Grx domains (Fig. 6) [126]. On the contrary, the Trx domain is not involved in any interaction with BOLA2. Chemical shift mapping identified well-defined interacting regions on both proteins, comprising the conserved His ligand of BOLA2 and the conserved Cys ligand of GLRX3 [126]. This apo complex is thus assembled in the proper structural arrangement to bind/receive two bridging [2Fe–2S]^2+^ clusters (Fig. 6). In human cells, a stable complex between GLRX3 and BOLA2 is observed only when they coordinate bridging [2Fe–2S] clusters [174]. The apo complex is not detected, possibly as a consequence of its lower stability with respect to the holo complex, which stabilizes the BOLA2–GLRX3 interaction by bridging two [2Fe–2S]^2+^ clusters between two BOLA2 molecules and each monothiol glutaredoxin domain of GLRX3 [126, 184]. However, the existence and a functional role of the apo complex at the cellular level cannot definitively be excluded. Which is the system donating [2Fe–2S]^2+^ clusters to the apo complex is still under investigation. A possible pathway, that has been recently proposed, involves the mitochondrial ISC assembly machinery: the de novo biosynthesis of the [2Fe–2S]^2+^ cluster on mitochondrial ISCU2 is followed by cluster export, as [2Fe–2S](GS)_4_ complex, via a membrane transporter; then, the [2Fe–2S] cluster is believed to be uptaken by the cytosolic form of ISCU2, which delivers it to cytosolic form of NFU1, which finally transfers the cluster to GLRX3 [189–192]. This molecular process is, however, only based on in vitro information, and experimental evidences from in vivo data are required to definitively validate it. Regardless of how GLRX3 acquires the [2Fe–2S] clusters, the functional role of the [2Fe–2S] GLRX3–BOLA2_2_ have been defined. NMR data showed that the [2Fe–2S]_2_ GLRX3–BOLA2_2_ complex in vitro transfers both its [2Fe–2S]^2+^ clusters to apo anamorsin producing its mature holo state, through the same protein–protein recognition mechanism operative in the GLRX3-anamorsin interaction, that specifically occurs between the N-terminal domains of the two proteins (Fig. 6) [126]. The [2Fe–2S]_2_ GLRX3–BOLA2_2_ complex maturing anamorsin cannot be formed via the interaction between BOLA2 and [2Fe–2S]_2_ GLRX3_2_, since NMR data showed that the formed heterotrimeric GLRX3–BOLA2_2_ complex contains only one [2Fe–2S]^2+^ cluster per complex. These in vitro data support a model where the apo heterotrimeric GLRX3–BOLA2_2_ complex is the species that, being able to acquire two [2Fe–2S] clusters, matures anamorsin [126]. UV–Vis CD data showing incomplete conversion from [2Fe–2S]_2_ GLRX3_2_ homodimer to [2Fe–2S]_2_ GLRX3–BOLA2_2_ heterotrimer are in agreement with this model [184]. These NMR data represent the first experimental evidence that the heterotrimeric GLRX3–BOLA2_2_ complex might work as a [2Fe–2S]^2+^ cluster transfer component in CIA machinery pathways. Accordingly with this proposal, a work in human cells reproduced these in vitro findings, showing that GLRX3–BOLA2_2_ complex delivers [2Fe–2S] clusters to anamorsin via a direct protein–protein interaction [174]. Collectively, in vitro and in vivo data showed that the GLRX3–BOLA2_2_ complex in mammalian cells functions as a [2Fe–2S] cluster chaperone, storing and delivering [2Fe–2S] clusters. It has been found that GLRX3_2_ homodimers represent a rare species of GLRX3 in cells with respect to BOLA2_2_–GLRX3 homotrimers [174], possibly because cluster binding is more labile and oxygen-sensitive in GLRX3_2_ than in GLRX3–BOLA2_2_ [184, 193]. Therefore, it cannot be definitively excluded that also [2Fe–2S]_2_ GLRX3_2_ works as [2Fe–2S] cluster chaperone in human cells.

In vivo data also showed that the iron bound to anamorsin did not completely disappear in cells lacking GLRX3 or BOLA2 [174]. This indicates that anamorsin may be capable of acquiring Fe–S clusters from an alternative source. It has been proposed that the mitoNEET/miner1 family of [2Fe–2S] proteins, which in vitro transfer their two [2Fe–2S] clusters to anamorsin [194], might be the alternative source of [2Fe–2S] clusters [195]. However, whether mitoNEET/miner1 proteins transfer [2Fe–2S] clusters to anamorsin in vivo is still unknown. So far, it has been only shown that mitoNEET can repair oxidatively damaged [4Fe–4S] clusters of iron regulatory protein 1 (IRP1) [195], a critical regulator of genes important for iron homeostasis and oxygen sensing [196]. This potential cluster transfer function of mitoNEET is linked to the redox state of the two [2Fe–2S] clusters bound to the protein, since, in their reduced state, the clusters are not released, while, in their oxidized state, the clusters can be transferred to apo proteins [197, 198]. A possible redox system regulating the cluster redox state of mitoNEET is composed by the cytosolic electron-donor NADPH/Ndor1/anamorsin complex, the component of the CIA machinery discussed above. NMR data showed that the [2Fe–2S] clusters of mitoNEET are reduced by anamorsin via the formation of a transient complex that brings the [2Fe–2S] clusters of mitoNEET close to the redox-active [2Fe–2S] cluster of anamorsin [199]. These data provide an in vitro evidence of a possible direct link between the CIA machinery and the mitoNEET-dependent repair pathway of IRP1: once oxidative stress is not occurring anymore in the cell, the Ndor1/anamorsin complex of the CIA machinery is functionally active in the cytoplasm [200] and can reduce the clusters of mitoNEET. In this way, the CIA machinery would stop the mitoNEET cluster transfer pathway repairing IRP1. The repair pathway is, indeed, no longer needed, once cellular oxidative stress is no more effective. In conclusion, the in vitro NMR data provided valuable input for testing, via cellular studies, whether a direct link between the CIA pathway and the mitoNEET-cluster transfer pathway exist in human cells.

## Conclusions

We believe that, in the coming years, solution NMR will be fundamental to describe the mechanisms through which [2Fe–2S] and [4Fe–4S] clusters are specifically transferred to the mitochondrial and cytosolic final targets. These processes are based on a concerted action of several accessory proteins, which have been clearly identified. However, the interaction networks among these accessory proteins are still quite elusive, and their molecular action is not clearly defined. We believe that solution NMR can contribute significantly to answer these questions for two main reasons. First, these interactions are expected to be transient and not permanent. Indeed, they need to be formed once the Fe–S cluster is delivered from the donor(s) to the final target, and then to be disrupted once the cluster has been transferred, thus producing a matured final target. Second, solution NMR is the optimal technique to investigate, at near-physiological conditions and at an atomic level, protein–protein interaction events that are transient and weak. Solution NMR will be able to clearly define the molecular function of these interactions, and thus open up new horizons on how to deal with the human diseases related to defects in Fe–S protein biogenesis processes, which have been growing rapidly. In this respect, we believe that a crucial breakthrough in the Fe–S protein field might be determined by the development and the application of paramagnetic NMR methods to characterize protein–protein complexes directly in mammalian cells. Indeed, it will be possible to study at atomic resolution the interactions responsible for maturing Fe–S proteins in living cells and to investigate physiological cluster-binding properties and cluster redox states.

## References

[1] Kowalsky A (1965). Biochemistry.

[2] Wüthrich K (1969). Proc Natl Acad Sci USA.

[3] McDonald CC, Phillips WD, Vinogradov SN (1969). Biochem Biophys Res Commun.

[4] Phillips WD, Poe M, Weiher JF, McDonald CC, Lovenberg W (1970). Nature.

[5] Poe M, Phillips WD, McDonald CC, Lovenberg W (1970). Proc Natl Acad Sci USA.

[6] Phillips WD, Poe M, McDonald CC, Bartsch RG (1970). Proc Natl Acad Sci USA.

[7] Dunham WR, Palmer G, Sands RH, Bearden AJ (1971). Biochim Biophys Acta.

[8] Saalmen I, Palmer G (1972). Arch Biochem Biophys.

[9] Poe M, Phillips WD, Glickson JD, McDonald CC, San Pietro A (1971). Proc Natl Acad Sci USA.

[10] Anderson RE, Dunham WR, Sands RH, Bearden AJ, Crespi HL (1975). Biochim Biophys Acta.

[11] Packer EL, Rabinowitz JC, Sternlicht H (1978). J Biol Chem.

[12] Werth MT, Kurtz DM, Moura I, LeGall J (1987). J Am Chem Soc.

[13] Xia B, Westler WM, Cheng H, Meyer J, Moulis J-M, Markley JL (1995). J Am Chem Soc.

[14] Goodfellow BJ, Tavares P, Romão MJ, Czaja C, Rusnak F, Le Gall J, Moura I, Moura JJG (1996). JBIC.

[15] Banci L, Bertini I, Luchinat C (1990). Struct Bond.

[16] Beinert H, Holm RH, Munck E (1997). Science.

[17] Noodleman L (1988). Inorg Chem.

[18] Blondin G, Girerd J-J (1990). Chem Rev.

[19] Machonkin TE, Westler WM, Markley JL (2005). Inorg Chem.

[20] Banci L, Bertini I, Briganti F, Luchinat C (1991). N J Chem.

[21] Dunham WR, Bearden AJ, Salmeen I, Palmer G, Sands RH, Orme-Johnson WH, Beinert H (1971). Biochim Biophys Acta.

[22] Banci L, Bertini I, Luchinat C (1991). Nuclear and electron relaxation. The magnetic nucleus-unpaired electron coupling in solution.

[23] Cheng H, Xia B, Reed GH, Markley JL (1994). Biochemistry.

[24] Orio M, Mouesca JM (2008). Inorg Chem.

[25] Dugad LB, La Mar GN, Banci L, Bertini I (1990). Biochemistry.

[26] Sheridan RP, Allen LC, Carter CWJ (1981). J Biol Chem.

[27] Xia B, Pikus JD, McClay K, Steffan RJ, Chae YK, Westler WM, Markley JL, Fox DJ (1999). Biochemistry.

[28] Skjeldal L, Westler WM, Oh B-H, Krezel AM, Holden HM, Jacobson BL, Rayment I, Markley JL (1991). Biochemistry.

[29] Huynh BH, Moura JJG, Moura I, Kent TA, LeGall J, Xavier AV, Münck E (1980). J Biol Chem.

[30] Kent TA, Huynh BH, Munk E (1980). Proc Natl Acad Sci USA.

[31] Bertini I, Ciurli S, Luchinat C (1995). Struct Bond.

[32] Cheng H, Grohmann K, Sweeney WV (1990). J Biol Chem.

[33] Busse SC, La Mar GN, Yu LP, Howard JB, Smith ET, Zhou ZH, Adams MWW (1992). Biochemistry.

[34] Macedo AL, Moura I, Moura JJG, LeGall J, Huynh BH (1993). Inorg Chem.

[35] Bentrop D, Bertini I, Luchinat C, Mendes J, Piccioli M, Teixeira M (1996). Eur J Biochem.

[36] Papaefthymiou V, Girerd J-J, Moura I, Moura JJG, Münck E (1987). J Am Chem Soc.

[37] Thomson AJ, Robinson AE, Johnson MK, Moura JJG, Moura I, Xavier AV, LeGall J (1981). Biochim Biophys Acta.

[38] Thompson CL, Johnson CE, Dickson DPE, Cammack R, Hall DO, Weser U, Rao KK (1974). Biochem J.

[39] Bertini I, Briganti F, Luchinat C, Scozzafava A (1990). Inorg Chem.

[40] Bertini I, Capozzi F, Ciurli S, Luchinat C, Messori L, Piccioli M (1992). J Am Chem Soc.

[41] Bertini I, Capozzi F, Luchinat C, Piccioli M, Vicens Oliver M (1992). Inorg Chim Acta.

[42] Bertini I, Capozzi F, Luchinat C, Piccioli M (1993). Eur J Biochem.

[43] Bertini I, Capozzi F, Luchinat C, Piccioli M, Vila AJ (1994). J Am Chem Soc.

[44] Bertini I, Donaire A, Feinberg BA, Luchinat C, Piccioli M, Yuan H (1995). Eur J Biochem.

[45] Backes G, Mino Y, Loehr TM, Meyer TE, Cusanovich MA, Sweeney WV, Adman ET, Sanders-Loehr J (1991). J Am Chem Soc.

[46] Langen R, Jensen GM, Jacob U, Stephen PJ, Warshel A (1992). J Biol Chem.

[47] Jensen GM, Warshel A, Stephen PJ (1994). Biochemistry.

[48] Perrin BS, Niu S, Ichiye T (2013). J Comput Chem.

[49] Agarwal A, Li D, Cowan JA (1995). Proc Natl Acad Sci USA.

[50] Bertini I, Borsari M, Bosi M, Eltis LD, Felli IC, Luchinat C, Piccioli M (1996). J Biol Inorg Chem.

[51] Mathews R, Charlton S, Sands RH, Palmer G (1974). J Biol Chem.

[52] Bertini I, Briganti F, Luchinat C, Messori L, Monnanni R, Scozzafava A, Vallini G (1991). FEBS Lett.

[53] Mouesca J-M, Noodleman L, Case DA, Lamotte B (1995). Inorg Chem.

[54] Le Pape L, Lamotte B, Mouesca J-M, Rius GJ (1997). J Am Chem Soc.

[55] Antanaitis BC, Moss TH (1975). Biochim Biophys Acta.

[56] Middleton P, Dickson DPE, Johnson CE, Rush JD (1980). Eur J Biochem.

[57] Nettesheim DG, Harder SR, Feinberg BA, Otvos JD (1992). Biochemistry.

[58] Banci L, Bertini I, Capozzi F, Carloni P, Ciurli S, Luchinat C, Piccioli M (1993). J Am Chem Soc.

[59] Bertini I, Gaudemer A, Luchinat C, Piccioli M (1993). Biochemistry.

[60] Banci L, Bertini I, Ciurli S, Ferretti S, Luchinat C, Piccioli M (1993). Biochemistry.

[61] Bominaar EL, Borshch SA, Girerd J-J (1994). J Am Chem Soc.

[62] Banci L, Bertini I, Eltis LD, Felli IC, Kastrau DHW, Luchinat C, Piccioli M, Pierattelli R, Smith M (1994). Eur J Biochem.

[63] Bertini I, Luchinat C, Piccioli M (2001). Methods Enzymol.

[64] Bertini I, Eltis LD, Felli IC, Kastrau DHW, Luchinat C, Piccioli M (1995). Chem Eur J.

[65] Bertini I, Couture MMJ, Donaire A, Eltis LD, Felli IC, Luchinat C, Piccioli M, Rosato A (1996). Eur J Biochem.

[66] Banci L, Bertini I, Dikiy A, Kastrau DHW, Luchinat C, Sompornpisut P (1995). Biochemistry.

[67] Bertini I, Dikiy A, Kastrau DHW, Luchinat C, Sompornpisut P (1995). Biochemistry.

[68] Bertini I, Donaire A, Luchinat C, Rosato A (1997). Proteins Struct Funct Genet.

[69] Aono S, Bentrop D, Bertini I, Donaire A, Luchinat C, Niikura Y, Rosato A (1998). Biochemistry.

[70] Davy SL, Osborne MJ, Moore GR (1998). J Mol Biol.

[71] Im S-C, Liu G, Luchinat C, Sykes AG, Bertini I (1998). Eur J Biochem.

[72] Goodfellow BJ, Macedo AL (1999). Ann Rep NMR Spectrosc.

[73] Goodfellow BJ, Macedo AL, Rodrigues P, Moura I, Wray V, Moura JJG (1999). J Biol Inorg Chem.

[74] Webert H, Freibert SA, Gallo A, Heidenreich T, Linne U, Amlacher S, Hurt E, Muhlenhoff U, Banci L, Lill R (2014). Nat Commun.

[75] Kudhair BK, Hounslow AM, Rolfe MD, Crack JC, Hunt DM, Buxton RS, Smith LJ, Le Brun NE, Williamson MP, Green J (2017). Nat Commun.

[76] Ramelot TA, Cort JR, Goldsmith-Fischman S, Kornhaber GJ, Xiao R, Shastry R, Acton TB, Honig B, Montelione GT, Kennedy MA (2004). J Mol Biol.

[77] Kim JH, Tonelli M, Kim T, Markley JL (2012). Biochemistry.

[78] Pastore C, Adinolfi S, Huynen MA, Rybin V, Martin S, Mayer M, Bukau B, Pastore A (2006). Structure.

[79] Xu X, Scanu S, Chung JS, Hirasawa M, Knaff DB, Ubbink M (2010). Biochemistry.

[80] Goodfellow BJ, Duarte IC, Macedo AL, Volkman BF, Nunes SG, Moura I, Markley JL, Moura JJ (2010). J Biol Inorg Chem.

[81] Uzarska MA, Nasta V, Weiler BD, Spantgar F, Ciofi-Baffoni S, Saviello MR, Gonnelli L, Muhlenhoff U, Banci L, Lill R (2016). Elife.

[82] Brancaccio D, Gallo A, Mikolajczyk M, Zovo K, Palumaa P, Novellino E, Piccioli M, Ciofi-Baffoni S, Banci L (2014). J Am Chem Soc.

[83] Pochapsky TC, Mei Ye X, Ratnaswamy G, Lyons TA (1994). Biochemistry.

[84] Lelong C, Sétif P, Bottin H, André F, Neumann J-M (1995). Biochemistry.

[85] Feng Y, Zhong N, Rouhier N, Hase T, Kusunoki M, Jacquot JP, Jin C, Xia B (2006). Biochemistry.

[86] Pochapsky TC, Jain NU, Kuti M, Lyons TA, Heymont J (1999). Biochemistry.

[87] Hatanaka H, Tanimura R, Katoh S, Inagaki F (1997). J Mol Biol.

[88] Mo H, Pochapsky SS, Pochapsky TC (1999). Biochemistry.

[89] Marg BL, Schweimer K, Sticht H, Oesterhelt D (2005). Biochemistry.

[90] Miura R, Ichikawa Y (1991). J Biol Chem.

[91] Xia B, Volkman BF, Markley JL (1998). Biochemistry.

[92] Bentrop D, Bertini I, Iacoviello R, Luchinat C, Niikura Y, Piccioli M, Presenti C, Rosato A (1999). Biochemistry.

[93] Bertini I, Luchinat C, Niikura Y, Presenti C (2000). Proteins Struct Funct Genet.

[94] Rodrigues PM, Macedo AL, Goodfellow BJ, Moura I, Moura JJ (2006). J Biol Inorg Chem.

[95] Gray HB, Ellis WR, Bertini I, Gray HB, Lippard SJ, Valentine JS (1994). Electron transfer. Bioinorganic chemistry.

[96] Jain NU, Tjioe E, Savidor A, Boulie J (2005). Biochemistry.

[97] Bertini I, Cowan JA, Luchinat C, Natarajan K, Piccioli M (1997). Biochemistry.

[98] Cheng H, Westler WM, Xia B, Oh BH, Markley JL (1995). Arch Biochem Biophys.

[99] Banci L, Bertini I, Calderone V, Ciofi-Baffoni S, Giachetti A, Jaiswal D, Mikolajczyk M, Piccioli M, Winkelmann J (2013). Proc Natl Acad Sci USA.

[100] Bermel W, Bertini I, Felli IC, Piccioli M, Pierattelli R (2006). Prog NMR Spectrosc.

[101] Machonkin TE, Westler WM, Markley JL (2002). J Am Chem Soc.

[102] Machonkin TE, Westler WM, Markley JL (2004). J Am Chem Soc.

[103] Kostic M, Pochapsky SS, Pochapsky TC (2002). J Am Chem Soc.

[104] Goodfellow BJ, Nunes SG, Rusnak F, Moura I, Ascenso C, Moura JJ, Volkman BF, Markley JL (2002). Protein Sci.

[105] Zartler ER, Jenney FE, Terrell M, Eidsness MK, Adams MW, Prestegard JH (2001). Biochemistry.

[106] Bertini I, Cavallaro G, Cosenza M, Kümmerle R, Luchinat C, Piccioli M, Poggi L (2002). J Biomol NMR.

[107] Piccioli M, Turano P (2015). Coord Chem Rev.

[108] Bertini I, Jiménez B, Piccioli M (2005). J Magn Reson.

[109] Ciofi-Baffoni S, Gallo A, Muzzioli R, Piccioli M (2014). J Biomol NMR.

[110] Zuiderweg ER (2002). Biochemistry.

[111] Perkins JR, Diboun I, Dessailly BH, Lees JG, Orengo C (2010). Structure.

[112] Lill R (2009). Nature.

[113] Maio N, Rouault TA (2015). Biochim Biophys Acta.

[114] Ciofi-Baffoni S, Nasta V, Banci L (2018). Metallomics.

[115] Dominguez C, Boelens R, Bonvin AM (2003). J Am Chem Soc.

[116] Nasta V, Giachetti A, Ciofi-Baffoni S, Banci L (2017). Biochim Biophys Acta.

[117] Yan R, Adinolfi S, Iannuzzi C, Kelly G, Oregioni A, Martin S, Pastore A (2013). PLoS One.

[118] Fuzery AK, Tonelli M, Ta DT, Cornilescu G, Vickery LE, Markley JL (2008). Biochemistry.

[119] Kim JH, Bothe JR, Alderson TR, Markley JL (2015). Biochim Biophys Acta.

[120] Prischi F, Pastore A (2017). Front Mol Biosci.

[121] Banci L, Ciofi-Baffoni S, Mikolajczyk M, Winkelmann J, Bill E, Eirini Pandelia M (2013). J Biol Inorg Chem.

[122] Musco G, Stier G, Kolmerer B, Adinolfi S, Martin S, Frenkiel T, Gibson T, Pastore A (2000). Structure.

[123] Cai K, Liu G, Frederick RO, Xiao R, Montelione GT, Markley JL (2016). Structure.

[124] Noguera ME, Aran M, Smal C, Vazquez DS, Herrera MG, Roman EA, Alaimo N, Gallo M, Santos J (2017). Arch Biochem Biophys.

[125] Li J, Ding S, Cowan JA (2013). Biochemistry.

[126] Banci L, Camponeschi F, Ciofi-Baffoni S, Muzzioli R (2015). J Am Chem Soc.

[127] Banci L, Ciofi-Baffoni S, Gajda K, Muzzioli R, Peruzzini R, Winkelmann J (2015). Nat Chem Biol.

[128] Banci L, Brancaccio D, Ciofi-Baffoni S, Del Conte R, Gadepalli R, Mikolajczyk M, Neri S, Piccioli M, Winkelmann J (2014). Proc Natl Acad Sci USA.

[129] Brancaccio D, Gallo A, Piccioli M, Novellino E, Ciofi-Baffoni S, Banci L (2017). J Am Chem Soc.

[130] Keizers PH, Mersinli B, Reinle W, Donauer J, Hiruma Y, Hannemann F, Overhand M, Bernhardt R, Ubbink M (2010). Biochemistry.

[131] Cai K, Frederick RO, Kim JH, Reinen NM, Tonelli M, Markley JL (2013). J Biol Chem.

[132] Cai K, Tonelli M, Frederick RO, Markley JL (2017). Biochemistry.

[133] Boniecki MT, Freibert SA, Muhlenhoff U, Lill R, Cygler M (2017). Nat Commun.

[134] Gakh O, Ranatunga W, Smith DY, Ahlgren EC, Al-Karadaghi S, Thompson JR, Isaya G (2016). J Biol Chem.

[135] Cory SA, Van Vranken JG, Brignole EJ, Patra S, Winge DR, Drennan CL, Rutter J, Barondeau DP (2017). Proc Natl Acad Sci USA.

[136] Muhlenhoff U, Gerber J, Richhardt N, Lill R (2003). EMBO J.

[137] Uzarska MA, Dutkiewicz R, Freibert SA, Lill R, Muhlenhoff U (2013). Mol Biol Cell.

[138] Bandyopadhyay S, Gama F, Molina-Navarro MM, Gualberto JM, Claxton R, Naik SG, Huynh BH, Herrero E, Jacquot JP, Johnson MK, Rouhier N (2008). EMBO J.

[139] Rodriguez-Manzaneque MT, Tamarit J, Belli G, Ros J, Herrero E (2002). Mol Biol Cell.

[140] Muhlenhoff U, Richter N, Pines O, Pierik AJ, Lill R (2011). J Biol Chem.

[141] Muhlenhoff U, Gerl MJ, Flauger B, Pirner HM, Balser S, Richhardt N, Lill R, Stolz J (2007). Eucaryotic Cell.

[142] Sheftel AD, Wilbrecht C, Stehling O, Niggemeyer B, Elsasser HP, Muhlenhoff U, Lill R (2012). Mol Biol Cell.

[143] Gelling C, Dawes IW, Richhardt N, Lill R, Muhlenhoff U (2008). Mol Cell Biol.

[144] Song D, Tu Z, Lee FS (2009). J Biol Chem.

[145] Tong WH, Jameson GN, Huynh BH, Rouault TA (2003). Proc Natl Acad Sci USA.

[146] Sheftel AD, Stehling O, Pierik AJ, Netz DJ, Kerscher S, Elsasser HP, Wittig I, Balk J, Brandt U, Lill R (2009). Mol Cell Biol.

[147] Melber A, Na U, Vashisht A, Weiler BD, Lill R, Wohlschlegel JA, Winge DR (2016). Elife.

[148] Blanc B, Gerez C, de Ollagnier CS (2015). Biochim Biophys Acta.

[149] Shi Y, Ghosh M, Kovtunovych G, Crooks DR, Rouault TA (2012). Biochim Biophys Acta.

[150] Sheftel AD, Stehling O, Pierik AJ, Elsasser HP, Muhlenhoff U, Webert H, Hobler A, Hannemann F, Bernhardt R, Lill R (2010). Proc Natl Acad Sci USA.

[151] Prudencio M, Ubbink M (2004). J Mol Recognit.

[152] Gray HB, Winkler JR (2003). Q Rev Biophys.

[153] Johansson C, Roos AK, Montano SJ, Sengupta R, Filippakopoulos P, Guo K, von Delft F, Holmgren A, Oppermann U, Kavanagh KL (2011). Biochem J.

[154] Bilder PW, Ding H, Newcomer ME (2004). Biochemistry.

[155] Cupp-Vickery JR, Silberg JJ, Ta DT, Vickery LE (2004). J Mol Biol.

[156] Morimoto K, Yamashita E, Kondou Y, Lee SJ, Arisaka F, Tsukihara T, Nakai M (2006). J Mol Biol.

[157] Wada K, Hasegawa Y, Gong Z, Minami Y, Fukuyama K, Takahashi Y (2005). FEBS Lett.

[158] Beilschmidt LK, de Ollagnier CS, Fournier M, Sanakis I, Hograindleur MA, Clemancey M, Blondin G, Schmucker S, Eisenmann A, Weiss A, Koebel P, Messaddeq N, Puccio H, Martelli A (2017). Nat Commun.

[159] Agar JN, Krebs C, Frazzon J, Huynh BH, Dean DR, Johnson MK (2000). Biochemistry.

[160] Chandramouli K, Unciuleac MC, Naik S, Dean DR, Huynh BH, Johnson MK (2007). Biochemistry.

[161] Jensen LT, Culotta VC (2000). Mol Cell Biol.

[162] Kaut A, Lange H, Diekert K, Kispal G, Lill R (2000). J Biol Chem.

[163] Andreini C, Banci L, Rosato A (2016). J Proteome Res.

[164] Stehling O, Netz DJ, Niggemeyer B, Rosser R, Eisenstein RS, Puccio H, Pierik AJ, Lill R (2008). Mol Cell Biol.

[165] Roy A, Solodovnikova N, Nicholson T, Antholine W, Walden WE (2003). EMBO J.

[166] Netz DJ, Pierik AJ, Stumpfig M, Muhlenhoff U, Lill R (2007). Nat Chem Biol.

[167] Stehling O, Vashisht AA, Mascarenhas J, Jonsson ZO, Sharma T, Netz DJ, Pierik AJ, Wohlschlegel JA, Lill R (2012). Science.

[168] Gari K, Leon Ortiz AM, Borel V, Flynn H, Skehel JM, Boulton SJ (2012). Science.

[169] Paul VD, Muhlenhoff U, Stumpfig M, Seebacher J, Kugler KG, Renicke C, Taxis C, Gavin AC, Pierik AJ, Lill R (2015). Elife.

[170] Stehling O, Mascarenhas J, Vashisht AA, Sheftel AD, Niggemeyer B, Rosser R, Pierik AJ, Wohlschlegel JA, Lill R (2013). Cell Metab.

[171] Muhlenhoff U, Molik S, Godoy JR, Uzarska MA, Richter N, Seubert A, Zhang Y, Stubbe J, Pierrel F, Herrero E, Lillig CH, Lill R (2010). Cell Metab.

[172] Haunhorst P, Hanschmann EM, Brautigam L, Stehling O, Hoffmann B, Muhlenhoff U, Lill R, Berndt C, Lillig CH (2013). Mol Biol Cell.

[173] Ojeda L, Keller G, Muhlenhoff U, Rutherford JC, Lill R, Winge DR (2006). J Biol Chem.

[174] Frey AG, Palenchar DJ, Wildemann JD, Philpott CC (2016). J Biol Chem.

[175] Hoffmann B, Uzarska MA, Berndt C, Godoy JR, Haunhorst P, Lillig CH, Lill R, Muhlenhoff U (2011). Antioxid Redox Signal.

[176] Li H, Outten CE (2012). Biochemistry.

[177] Haunhorst P, Berndt C, Eitner S, Godoy JR, Lillig CH (2010). Biochem Biophys Res Commun.

[178] Saito Y, Shibayama H, Tanaka H, Tanimura A, Matsumura I, Kanakura Y (2011). Biochem Biophys Res Commun.

[179] Soler N, Delagoutte E, Miron S, Facca C, Baille D, d’Autreaux B, Craescu G, Frapart YM, Mansuy D, Baldacci G, Huang ME, Vernis L (2011). Mol Microbiol.

[180] Netz DJ, Stumpfig M, Dore C, Muhlenhoff U, Pierik AJ, Lill R (2010). Nat Chem Biol.

[181] Banci L, Bertini I, Ciofi-Baffoni S, Boscaro F, Chatzi A, Mikolajczyk M, Tokatlidis K, Winkelmann J (2011). Chem Biol.

[182] Zhang Y, Yang C, Dancis A, Nakamaru-Ogiso E (2017). J Biochem.

[183] Netz DJ, Genau HM, Weiler BD, Bill E, Pierik AJ, Lill R (2016). Biochem J.

[184] Li H, Mapolelo DT, Randeniya S, Johnson MK, Outten CE (2012). Biochemistry.

[185] Vilella F, Alves R, Rodriguez-Manzaneque MT, Belli G, Swaminathan S, Sunnerhagen P, Herrero E (2004). Comp Funct Genom.

[186] Huynen MA, Spronk CA, Gabaldon T, Snel B (2005). FEBS Lett.

[187] Zhou YB, Cao JB, Wan BB, Wang XR, Ding GH, Zhu H, Yang HM, Wang KS, Zhang X, Han ZG (2008). Mol Cell Biochem.

[188] Kumanovics A, Chen OS, Li L, Bagley D, Adkins EM, Lin H, Dingra NN, Outten CE, Keller G, Winge D, Ward DM, Kaplan J (2008). J Biol Chem.

[189] Wachnowsky C, Fidai I, Cowan JA (2016). FEBS Lett.

[190] Li J, Cowan JA (2015). Chem Commun (Camb).

[191] Qi W, Li J, Chain CY, Pasquevich GA, Pasquevich AF, Cowan JA (2012). J Am Chem Soc.

[192] Qi W, Li J, Cowan JA (2014). Chem Commun (Camb).

[193] Nuttle X, Giannuzzi G, Duyzend MH, Schraiber JG, Narvaiza I, Sudmant PH, Penn O, Chiatante G, Malig M, Huddleston J, Benner C, Camponeschi F, Ciofi-Baffoni S, Stessman HA, Marchetto MC, Denman L, Harshman L, Baker C, Raja A, Penewit K, Janke N, Tang WJ, Ventura M, Banci L, Antonacci F, Akey JM, Amemiya CT, Gage FH, Reymond A, Eichler EE (2016). Nature.

[194] Lipper CH, Paddock ML, Onuchic JN, Mittler R, Nechushtai R, Jennings PA (2015). PLoS One.

[195] Ferecatu I, Goncalves S, Golinelli-Cohen MP, Clemancey M, Martelli A, Riquier S, Guittet E, Latour JM, Puccio H, Drapier JC, Lescop E, Bouton C (2014). J Biol Chem.

[196] Rouault TA, Klausner RD (1996). Trends Biochem Sci.

[197] Zuris JA, Harir Y, Conlan AR, Shvartsman M, Michaeli D, Tamir S, Paddock ML, Onuchic JN, Mittler R, Cabantchik ZI, Jennings PA, Nechushtai R (2011). Proc Natl Acad Sci USA.

[198] Golinelli-Cohen MP, Lescop E, Mons C, Goncalves S, Clemancey M, Santolini J, Guittet E, Blondin G, Latour JM, Bouton C (2016). J Biol Chem.

[199] Camponeschi F, Ciofi-Baffoni S, Banci L (2017). J Am Chem Soc.

[200] Vernis L, Facca C, Delagoutte E, Soler N, Chanet R, Guiard B, Faye G, Baldacci G (2009). PLoS One.

[201] Xia B, Jenk D, LeMaster DM, Westler WM, Markley JL (2000). Arch Biochem Biophys.

