# Correction to: The NMR contribution to protein–protein networking in Fe–S protein maturation

**DOI:** 10.1007/s00775-018-1573-5

**Published:** 2018-05-31

**Authors:** Lucia Banci, Francesca Camponeschi, Simone Ciofi‑Baffoni, Mario Piccioli

**Affiliations:** 1 Magnetic Resonance Center CERM, University of Florence, Via Luigi Sacconi 6, Sesto Fiorentino, 50019 Florence, Italy; 2 Department of Chemistry, University of Florence, Via della Lastruccia 3, Sesto Fiorentino, 50019 Florence, Italy

## Correction to: JBIC Journal of Biological Inorganic Chemistry 10.1007/s00775-018-1552-x

The article “The NMR contribution to protein–protein networking in Fe–S protein maturation”, written by Lucia Banci, Francesca Camponeschi, Simone Ciofi-Baffoni, Mario Piccioli was originally published electronically on the publisher’s internet portal (currently SpringerLink) without open access.

The copyright of the article changed on 29, May to © The Author(s) 2018 and the article is forthwith distributed under the terms of the Creative Commons Attribution 4.0 International License (http://creativecommons.org/licenses/by/4.0/), which permits use, duplication, adaptation, distribution and reproduction in any medium or format, as long as you give appropriate credit to the original author(s) and the source, provide a link to the Creative Commons license and indicate if changes were made.

The original article has been corrected.

